# Machine learning approaches identify male body size as the most accurate predictor of species richness 

**DOI:** 10.1186/s12915-020-00835-y

**Published:** 2020-08-28

**Authors:** Klemen Čandek, Urška Pristovšek Čandek, Matjaž Kuntner

**Affiliations:** 1 Evolutionary Zoology Laboratory, Department of Organisms and Ecosystems Research, National Institute of Biology, Ljubljana, Slovenia; 2 Jovan Hadži Institute of Biology, Research Centre of the Slovenian Academy of Sciences and Arts, Ljubljana, Slovenia; 3 Department of Biology, Biotechnical Faculty, University of Ljubljana, Ljubljana, Slovenia; 4 State Key Laboratory of Biocatalysis and Enzyme Engineering, Centre for Behavioural Ecology and Evolution, School of Life Sciences, Hubei University, Wuhan, Hubei China; 5 Department of Entomology, National Museum of Natural History, Smithsonian Institution, Washington D.C., USA

**Keywords:** Biodiversity, Lineage diversity, Species traits, Spiders, Phylogenetic diversity, Species distribution, Random Forest, Multiple correspondence analysis

## Abstract

**Background:**

A major challenge in biodiversity science is to understand the factors contributing to the variability of species richness –the number of different species in a community or region - among comparable taxonomic lineages. Multiple biotic and abiotic factors have been hypothesized to have an effect on species richness and have been used as its predictors, but identifying accurate predictors is not straightforward. Spiders are a highly diverse group, with some 48,000 species in 120 families; yet nearly 75% of all species are found within just the ten most speciose families. Here we use a Random Forest machine learning algorithm to test the predictive power of different variables hypothesized to affect species richness of spider genera.

**Results:**

We test the predictive power of 22 variables from spiders’ morphological, genetic, geographic, ecological and behavioral landscapes on species richness of 45 genera selected to represent the phylogenetic and biological breath of Araneae. Among the variables, Random Forest analyses find body size (specifically, minimum male body size) to best predict species richness. Multiple Correspondence analysis confirms this outcome through a negative relationship between male body size and species richness. Multiple Correspondence analyses furthermore establish that geographic distribution of congeneric species is positively associated with genus diversity, and that genera from phylogenetically older lineages are species poorer. Of the spider-specific traits, neither the presence of ballooning behavior, nor sexual size dimorphism, can predict species richness.

**Conclusions:**

We show that machine learning analyses can be used in deciphering the factors associated with diversity patterns. Since no spider-specific biology could predict species richness, but the biologically universal body size did, we believe these conclusions are worthy of broader biological testing. Future work on other groups of organisms will establish whether the detected associations of species richness with small body size and wide geographic ranges hold more broadly.

## Background

The search for general mechanisms responsible for the observed differences in biodiversity patterns across the tree of life is the focus of many areas of biological research [1–4]. Detecting such mechanisms would enable predictions of species richness by proxies and would be important in ecology, biogeography, evolution, and conservation biology [5]. Variation in species richness among lineages of comparable taxonomic ranks is often studied locally or within an island system [6, 7]. The often detected discrepant patterns are primarily explained by variation in organismal dispersal ability [8, 9], niche preemption [10], habitat complexity [11], and the time since a given lineage has occupied the studied area [12]. On the other hand, the identification of attributes impacting large-scale species richness variation, and the extent of its effect, remains opaque and would require more complex approaches [13, 14].

One of the fundamental questions in biology remains to be adequately addressed. Namely, what factors might contribute to the high variation in biodiversity among comparable taxonomic lineages? Multiple biotic and abiotic factors have been hypothesized to have an effect on species richness, and a few of them have been used as its predictors [15–18]. For example, several studies associate high species richness with variables that correlate with small body size [19–21]. More directly, body size has been used as a predictor of total species richness in beetles and wider [22]. Other studies have been unable to detect a correlation between species richness and body size, let alone imply causality [23–25]. Further organismal attributes have been proposed to explain the variation in biodiversity among lineages, most prominently an organism’s generation time, and clade age. A shorter generation time generally correlates with a higher rate of DNA mutation accumulation and should in theory lead to higher species richness ([26, 27] but see [28]). Similarly, phylogenetically older clades are sometimes linked with higher biodiversity due to longer time available for speciation ([29–31] but see [3, 32]). Dispersal abilities and intrinsic lineage tendencies for speciation have a notable effect on the creation of discrepancies in species richness among genera or other comparable taxonomic ranks [8, 33, 34]. The effect of the dispersal ability of a given lineage on its species richness, however, is most likely not linear [9, 35].

Additional hypotheses predict that ecological opportunity [28] and shifts in species ecology and behavior significantly affect species richness. Specializations in e.g. distinct feeding strategies [23], mating systems and associated phenotypes [36], or even secondary loss of dispersal abilities [34, 37] have all been linked with increases in species richness following adaptive radiation. However, specialization is sometimes associated with higher extinction rates and considered an evolutionary dead-end, decreasing biodiversity of lineages [38, 39]. Furthermore, abiotic factors such as geographic range of taxa [40], habitat complexity and fragmentation [6, 41], climate [42–44], or the presence of archipelagos [6, 45] can all affect speciation or extinction rates, resulting in varying degrees of biodiversity among lineages. Some studies have predicted the total species richness from the proportion of endemic species [46, 47] or from rare and indicator species [48]. Finally, genetic diversity, while better researched in association with geographic distribution and geographic isolation of taxa [49], does correlate with species richness ([50, 51] but see [52]).

The task of identifying good predictors for species richness among a large number of variables requires powerful analytical tools. From the list of the above described predictor variables, many observations can only be classified as categorical or binary data. Others are frequencies or continuous numerical data. Such mixed types of variables can be difficult to analyze simultaneously, and within a single statistic. Machine or ensemble learning statistic methods, more specifically the Random Forest [53] ensemble learning algorithm, can handle such mixed data. Random Forest (RF) operates by “growing” multiple Decision Trees [54], yet another machine learning algorithm capable of fitting complex datasets and performing both classification and regression tasks. Decision trees “learn” from the training dataset (usually a random selection of about 70% of rows in a matrix) to predict the outcome for the new data. Random Forest grows multiple decision trees and uses bootstrap aggregating as well as a random subset of predictor variables to grow them. Therefore, RF greatly improves the predicted outcome, compared to a single decision tree [55]. Random Forest recovers the most important features/predictors by analyzing the “votes” of decision trees. These important predictors are more closely related to the dependent variable and contribute more towards explaining its total variability. However, even robust algorithms like RF are sensitive to intense “noise” in the data; thus, carefully choosing the right predictor variables can make the RF prediction model more accurate.

Biodiversity science profits most from studying global patterns in species-rich taxonomic groups. Spiders represent one such lineage with a high taxonomic, ecological, and spatial variability in biodiversity among comparable subclades and geographic units. With 48,366 extant species grouped in 4152 genera and 120 families [56], spiders are truly megadiverse. As a large proportion of spider species are yet unknown, and many are extinct [57], estimates of true spider species richness range up to 170 thousand [58]. Considering the known taxonomic diversity, each family and genus, on average, contain roughly 403 and 12 species, respectively. The biological truth, however, is much more skewed, as 10% of the most speciose families comprise 73% of all species. Moreover, numerous genera are monotypic while others contain hundreds of species. These observed discrepancies in species richness among comparable taxonomic ranks of spiders are likely to be real even when considering the unknown portion of the diversity. Arriving at credible biological explanations for such skewness in biodiversity would be highly revealing.

Here, we focus on identifying the best predictor(s) for species richness in spider genera. Considering our comprehensive review of recent literature and the availability of data in public repositories, we select a combination of morphological, geographic, genetic, and behavioral–ecological variables to predict diversity patterns. Our set of predictor variables reflects spider biology as understood. For example, the average body size for female spiders is 6.9 mm, and for males is 5.7 mm [59], but spider body size ranges from microscopic (0.37 mm in *Patu digua*) to dinner-plate (119 mm in *Theraphosa blondi*). While the vast majority of spider species are relatively sexually size monomorphic, some selected subclades have evolved extreme levels of female-biased sexual size dimorphism that not only affects species biology [60, 61], but may also influence speciation and extinction [59]. Spider species and genus distributions span from endemic to cosmopolitan, and genetic data have become routinely available. Spiders exhibit numerous behavioral, ecological, and morphological specializations [62]. Moreover, spiders show varying dispersal potential, e.g., some species readily disperse long distances via rafting on silk (ballooning) while others do not [63], and these differences affect gene flow and genetic diversity [64]. In order to probe into the question of how such phenotypic, ecological, and genetic variables may influence species diversity, we assemble such data for 45 spider genera that we selected to represent the phylogenetic breadth of Araneae, and analyze them using RF ensemble learning algorithm. We then employ multiple correspondence analysis (MCA) to further expose the relationship between predictor variables and species richness and to compare those with RF predictions.

## Results

### Summary results

**Fig. 1:**
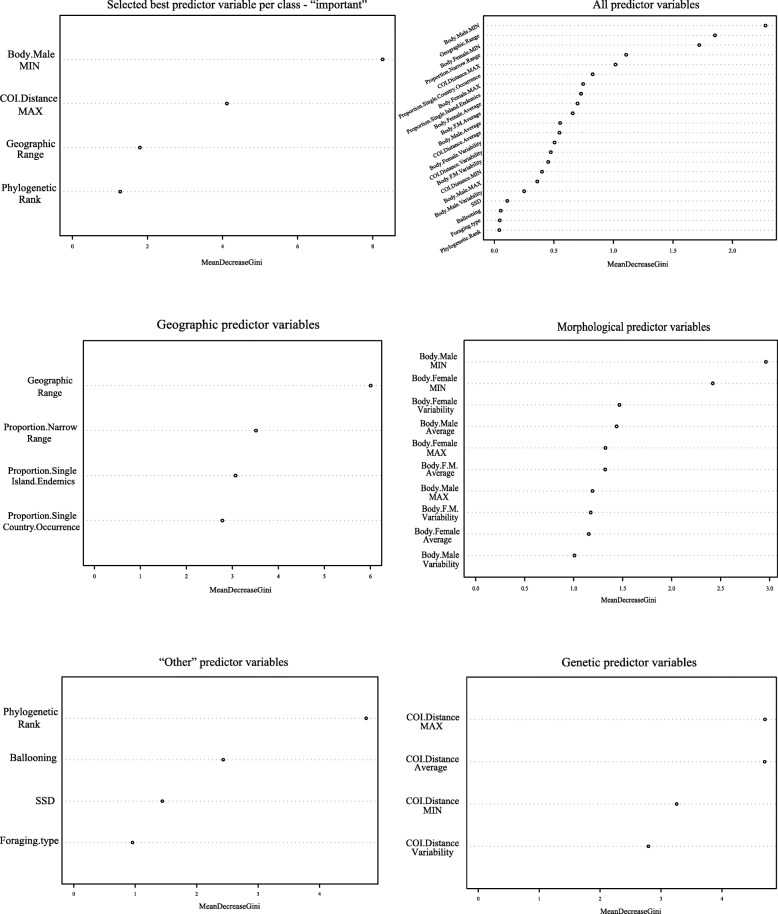
Random Forest (RF) results for different groups of variables. The bottom and middle panels represent RF results for specific groups of predictor variables. The top right panel represents RF results for the combination of all predictor variables while the top left panel shows RF results for the best predictor variables from each variable group only. Note that the greater Gini decrease for a specific variable is, the greater predictive power that variable has for species richness of spider genera

The RF models with the highest accuracy of classification for our dataset operate with two species richness categories defined as “small” and “high.” We therefore focus most on interpreting these results, but also present results from other RF models with more species richness categories, as well as RF regression models, in the supporting materials. What is common to all analyses using RF models is that they all recover minimum male body size as the best predictor of species richness whenever this variable is included (Fig. 1, Additional file 1: Figs. S1 – S4).

**Fig. 2:**
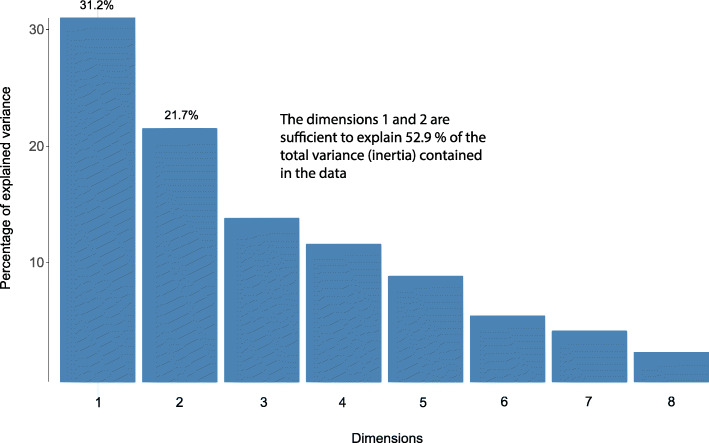
Histogram of the percent of total variance each MCA dimension is able to explain. MCA recovers the total of eight dimensions to explain the total variance contained in the dataset. However, to be able to visually interpret the results, we must reduce the number of dimensions. An interpretable graphic representation of MCA results best operates in two dimensions. Therefore, we relied on the first two MCA dimensions for investigating variable relationships. Those two dimensions together explain 52.9% of the total variance (inertia) within the data while the 47.1% of the variance is lost

**Fig. 3:**
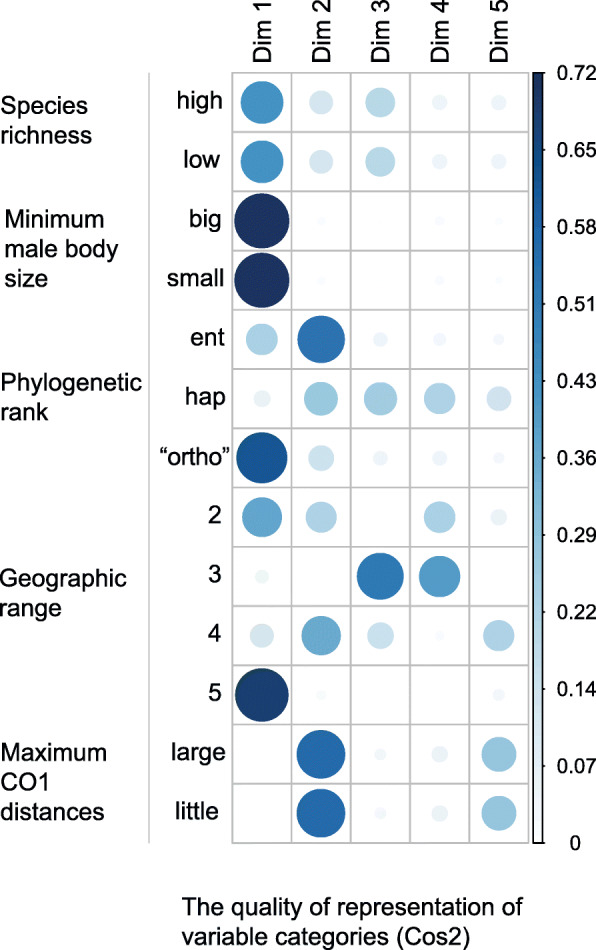
The quality of representation of variable categories within the first five MCA dimensions. Larger and darker circles represent better quality of representation of a variable category within a specific dimension using squared cosine or the squared correlations (cos2) values. Note that most of the variables are well represented in the first two dimensions (but see “Geographic range 3”)

**Fig. 4:**
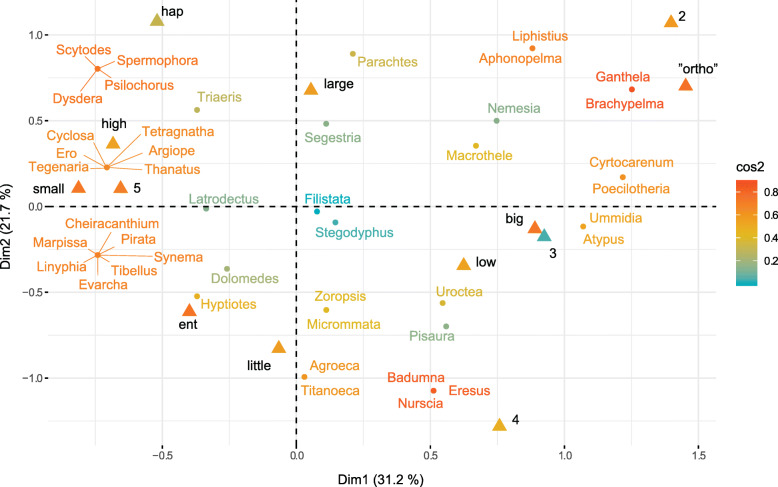
Biplot, combining individuals (dots) and variable categories (triangles) in two MCA dimensions. The color gradient indicates the cos2 quality of representation for each data point. The distance between any two points on the biplot is a measure of dissimilarity between them. Therefore, data with similar “profile” are closer together on the map (e.g., triangles for “high” species richness, “small” body size, “5” geographic range)

**Fig. 5:**
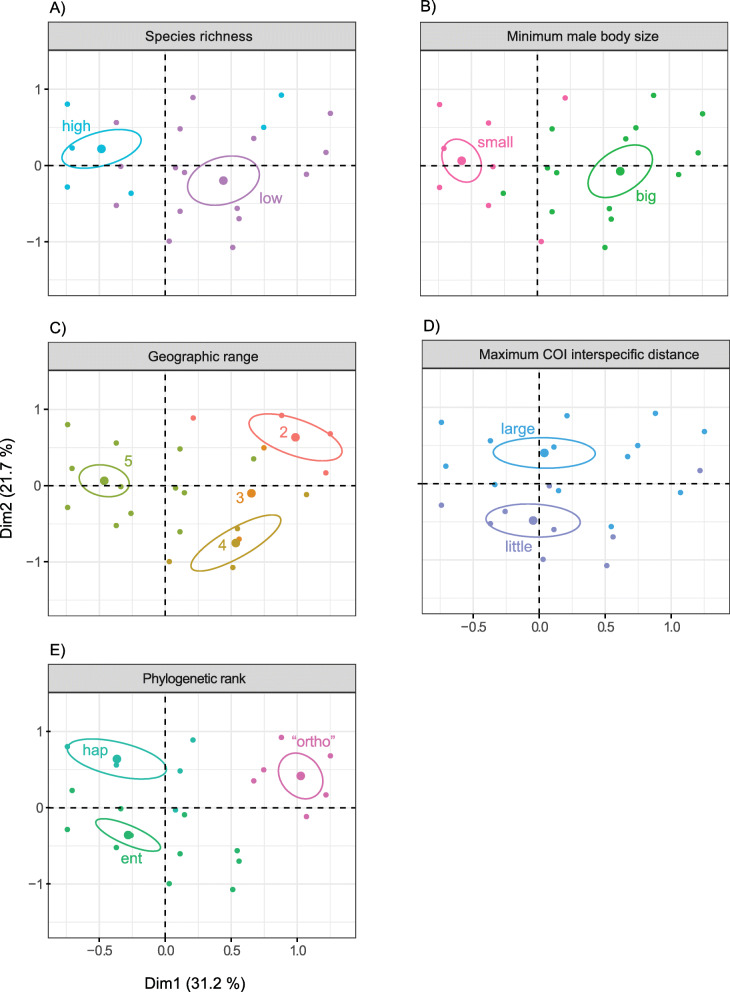
MCA factor map for classes of variables. In the MCA factor map, each panel (**a**–**e**) represents a class of variable. Within the panel, each individual point is colored by its variable category and each ellipse represents the confidence interval for the positioning of the variable category within the two MCA dimensions. This allows for the visual comparison of “profiles” among variable categories. Note the very similar “profiles” of the “high” species richness, “small” body size, and “5” geographic range, implying relationship among these variable categories

**Fig. 6:**
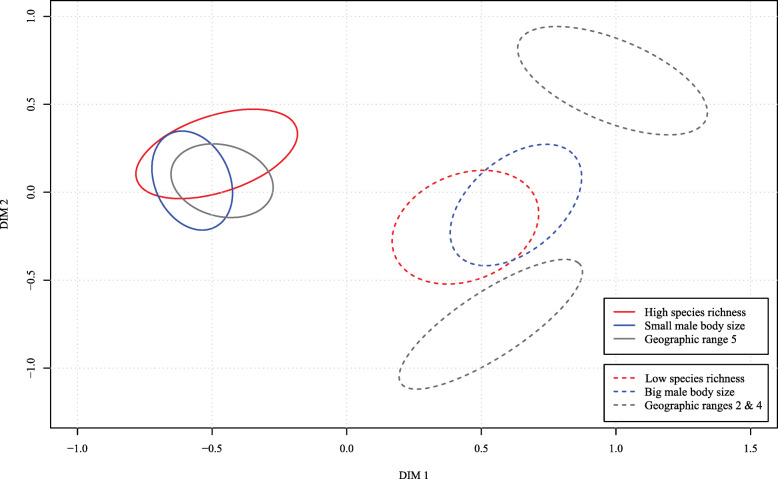
Overlaps of the confidence ellipses. This single plot combines confidence ellipses for the most relevant variable categories. Significant overlaps exist among high species richness, wide geographic distribution (range 5—cosmopolitan distribution), and small male body size in the two dimensions of the MCA. Note also a large overlap between confidence ellipses for big male body size and low species richness **41.82 %** of the **high species richness** confidence ellipse area overlaps with the **small male body size** confidence ellipse **69.56 %** of the **small male body size** confidence ellipse area overlaps with the **high species richness** confidence ellipse **36.99 %** of the **high species richness** confidence ellipse area overlaps with the **geographic range 5** confidence ellipse **64.33 %** of the **geographic range 5** confidence ellipse area overlaps with the **high species richness** confidence ellipse **54.22 %** of the **small male body size** confidence ellipse area overlaps with the **geographic range 5** confidence ellipse **56.68 %** of the **geographic range 5** confidence ellipse area overlaps with the **small male body size** confidence ellipse **50.53 %** of the** low species richness** confidence ellipse area overlaps with the **big male body size** confidence ellipse **57.65 %** of the **big male body size** confidence ellipse area overlaps with the **low species richness** confidence ellipse

MCA investigates the relations among five categorical variables: minimum male body size, maximum COI genetic distances, geographic range, phylogenetic rank, and species richness. Among the six different combinations of variable category definitions, the MCA with two species richness, two minimum male body size, and two maximum COI genetic distances categories explains the highest proportion of total variability (inertia) (Fig. 2) and has the best cos2 quality of representation (Fig. 3) of variable categories in the first two MCA dimensions. We therefore focus on interpreting the results of the “two categories” MCA, but present alternative MCA results in the supporting materials. In support of the RF analyses, all MCA analyses detect minimum male body size to associate with species richness regardless of species richness categorization, but additionally recover wide geographic range to also associate with high species richness (Figs. 4, 5, and 6, Additional file 1: Figs. S5 – S9).

### Random Forest classification with two species richness categories

#### All variables

RF analysis using all predictor variables for species richness recovers minimum male body size, followed by geographic range and minimum female body size as the best predictor variables. The optimized RF model on the training dataset uses six variables randomly sampled at each split when creating the tree models (mtry = 6) and grows 1000 decision trees. The estimated out of the bag (OOB) error is 18.75% while the actual accuracy when applying this model to the test dataset is 46.15% (Fig. 1).

#### Morphological variables

Using only morphological predictor variables for species richness, RF recovers minimum male body size as the best predictor. The optimized RF model on the training dataset uses three variables randomly sampled at each split when creating the tree models (mtry = 3) and grows 1000 decision trees. The estimated OOB error is 28.12% while the actual accuracy when applying this model to the test dataset is 46.15% (Fig. 1).

#### Genetic variables

RF using genetic predictor variables recovers maximum COI interspecific distances as the best predictor for species richness within this group. The optimized RF model on the training dataset uses two variables randomly sampled at each split when creating the tree models (mtry = 2) and grows 1000 decision trees. The estimated OOB error is 37.5% while the actual accuracy when applying this model to the test dataset is 53.84% (Fig. 1).

#### Geographic variables

RF using geographic predictor variables for species richness recovers geographic range as the best predictor within this group. The optimized RF model on the training dataset uses three variables randomly sampled at each split when creating the tree models (mtry = 3) and grows 1000 decision trees. The estimated OOB error is 28.12% while the actual accuracy when applying this model to the test dataset is 53.84% (Fig. 1).

#### “Other” variables

RF using the remaining variables: sexual size dimorphism (SSD), presence of ballooning, phylogenetic rank, and foraging type, grouped in “other” variable category, recovers phylogenetic rank as the best predictor for species richness within this group. The optimized RF model on the training dataset uses three variables randomly sampled at each split when creating the tree models (mtry = 3) and grows 1000 decision trees. The estimated OOB error is 34.38% while the actual accuracy when applying this model to the test dataset is 38.46% (Fig. 1).

#### Selected best predictors per group

RF using a single best predictor within each group of variables (“important,” favored by preceding RF analyses) recovers minimum male body size as the best predictor for species richness, followed by maximum COI genetic distances, geographic range, and phylogenetic rank. The optimized RF model on the training dataset uses three variables randomly sampled at each split when creating the tree models (mtry = 3) and grows 1000 decision trees. The estimated OOB error is 18.75% while the actual accuracy when applying this model to the test dataset is 69.23% (Fig. 1).

### Other Random Forest models and Spearman’s correlation

RF models operating with three species richness categories (“high,” “medium,” and “low”), using all predictor variables as well as only a single best predictor within each group of variables, both recover minimum male body size as the best predictor for species richness. The estimated OOB errors are 50% and 53.3%, respectively, and the actual accuracy when applying these models to the test datasets is 20% and 33.3%, respectively (Additional file 1: Figs. S1 and S2).

Regression models of RF that operate with species richness as a numeric variable, using all predictor variables as well as only a single best predictor within each group of variables, both recover minimum male body size as the best predictor for species richness (Additional file 1: Figs. S3 and S4). The percent of variance explained by these two RF regression models is 11.16 and 18.2, respectively.

Minimum male body size predictor variable and species richness were both non-normally distributed (Shapiro–Wilk normality test: minimum male body size *W* = 0.59, *p* < 0.001; species richness *W* = 0.71, *p* < 0.001; Additional file 1: Fig. S10). Spearman’s rank correlation analysis between these two variables detects a weak but significant negative association of high species richness and small male body size (rho = − 0.41, *p* = 0.005; Additional file 1: Fig. S10).

### Multiple correspondence analysis (MCA)

#### The “two categories” MCA

The “two categories” MCA (hereforth “MCA”) recovers eight dimensions to explain the total variability of the data (Fig. 2). Of those eight, the first two dimensions explain 52.9% of the total variability in the data (Dim 1 explains 31.2%; Dim 2 explains 21.7%). However, not all points are equally well represented in those two dimensions. The quality of representation, squared cosine or squared correlations (cos2), of the categories measures the degree of association between variable categories and a particular axis (dimension). The cos2 for our data (Fig. 3) shows a good representation of most variable categories in the first two dimensions. Cos2 is a relative value; therefore, the sum of a row in cos2 factor map (Fig. 3) is equal to one.

The MCA biplot (Fig. 4) shows a global pattern within the data. Rows (individuals) are depicted by points while columns (variable categories) are represented by triangles. The color gradient describes the cos2 quality of representation for both, individuals and variable categories in the two dimensions. Note that a few points, namely geographic range 3, *Stegodyphus* and *Filistata*, are not very well represented by the first two MCA dimensions. Therefore, the position of those points should be interpreted with some caution. The distance between any row points or between any column points gives a measure of their similarity (or dissimilarity). Distances between row and column points are usually incomparable due to their mathematical properties [65]; therefore, to make them comparable within the same plot, we transformed the row points to correctly reflect the column points with “map = rowprincipal” argument in “fviz_mca_biplot” function. Row points with similar profiles are merged on the biplot.

The MCA biplot (Fig. 4) combined with Table 1 suggests to which pole of the dimensions the row (individuals) and column (variable categories) points actually contribute. For example, it is evident that small male body size, high species richness, and broad geographic distribution (range 5) all contribute to the negative pole of DIM1, while big male body size, low species richness, and “ortho” phylogenetic rank contribute to the positive pole of DIM1. Similarly, “hap” phylogenetic rank and large COI genetic distances contribute to the positive pole of DIM2 while low COI distances and “ent” rank contribute to the negative pole of DIM2. Moreover, we can observe highly similar profiles for small male body size, high species richness, and broad geographic distribution (range 5). On the other hand, Entelegyne spiders appear to have lower maximum COI distances compared to the other two phylogenetic ranks (“hap” and “ortho”).

**Table 1:** Coordinates of variable categories on the first and on the second dimension of the MCA (DIM1 and DIM2)

Variable category	DIM1 coordinates	Variable category	DIM2 coordinates
**Small** body size	− 0.813	Geographic range **4**	− 1.274
**High** species richness	− 0.684	**Little** COI distances	− 0.821
Geographic range **5**	− 0.655	**Entelegynae** phylogenetic rank	− 0.606
**Haplogynae** phylogenetic rank	− 0.519	**Low** species richness	− 0.338
**Entelegynae** phylogenetic rank	− 0.399	Geographic range **3**	− 0.17
**Little** COI distances	− 0.065	**Big** body size	− 0.123
**Large** COI distances	0.055	Geographic range **5**	0.111
**Low** species richness	0.624	**Small** body size	0.112
Geographic range **4**	0.758	**High** species richness	0.37
**Big** body size	0.89	**Large** COI distances	0.684
Geographic range **3**	0.925	**“Orthognatha”** group	0.708
Geographic range **2**	1.398	Geographic range **2**	1.077
**“Orthognatha”** group	1.452	**Haplogynae** phylogenetic rank	1.086

The above patterns stand out on a MCA factor map (Fig. 5) and on a plot with the overlapping variable category confidence ellipses (Fig. 6). Each panel of Fig. 5 represents a class of variable: (A) species richness, (B) minimum male body size, (C) geographic range, (D) maximum COI genetic distance, and (E) phylogenetic rank. Each panel contains the variable category (column points) with confidence ellipse. The individuals (row points) are colored according to the variable category they represent in each panel. Here (Fig. 5), the relatedness of small male body size, high species richness, and broad geographic distribution (range 5) becomes apparent through visual assessment of their highly similar confidence ellipse profiles on the MCA factor map. Moreover, big maximum male body size and low species richness exhibit a similar profile, while narrow geographic range correlates with “ortho” phylogenetic rank. While confidence ellipses on a MCA factor map are usually only visually assessed, we augmented their interpretability by combining them within a single plot (Fig. 6) and by calculating the overlaps between pairs of ellipses. Large overlaps indicate a strong correlation among variable categories.

#### Other MCA

The other five MCA with alternative category definitions (categories ranging from two to five species richness categories, from two to five minimum male body sizes, and from two to three maximum COI genetic distance) served as a method sensitivity test. Results across all combinations were consistent in showing very similar profiles (and high overlaps) among small (or very small) male body size, high (or very high) species richness, and geographic range 5 (Additional file 1: Figs. S5 – S9). The same holds true at the other extreme with big (or very big) male body size profiles overlapping with the profiles of high (or very high) species richness. These sensitivity tests reinforce the above reported variable category associations. However, partitioning the data into many categories caused the cos2 quality of representation of some variable categories to drop within the first two MCA dimensions (Additional file 1: Figs. S5 – S9, panels “D”). Moreover, MCA with overpartitioned data explains less of the total variance contained within the dataset (Additional file 1: Figs. S5 – S8, panels “A”).

## Discussion

In the search for the best predictor of species richness in 45 spider genera that represent a compromise between the phylogenetic and biological breath of spiders and the available data, we assess 22 potential predictors from their morphological, genetic, geographic, ecological, and behavioral landscapes. RF analyses suggest that body size, or more specifically, the minimum male body size, predicts species richness best. The results from the MCA analyses confirm this RF outcome by recovering a negative relationship between male body size and species richness. Moreover, MCA suggests that a wide geographic distribution of congeneric species is positively associated with higher genus diversity. These results also show that genera from phylogenetically older groups of spiders are species poorer. Somewhat surprisingly, given the nuances of spider biology, we find that ballooning and sexual size dimorphism cannot predict species richness. While the detected association among variables does not imply causality, our results nonetheless find a certain predictability of species richness patterns.

Small body sizes are sometimes correlated with higher species richness of a clade [20, 21], a pattern also recovered here (Figs. 1, 4, 5, and 6, Additional file 1: all figs.). Arguably, certain features that co-vary with size, if not organismal size itself, might be the critical drivers of variability in species richness among lineages of comparable ranks. Such factors that relate with small body size include higher metabolic rates [18, 66], higher reproductive rates [67], the need for fewer resources [21], or the combination of limited dispersal capabilities, low physiological tolerances, and consequentially more fragmented ranges of smaller organisms [19, 68]. At least the latter combination of factors is highly unlikely in spiders as genera with small representatives readily disperse with ballooning and are commonly distributed over broad geographic ranges [62, 63, 69]. Our analyses suggest that the minimum male body size within a genus best predicts species richness, but this feature does correlate with most other body size variables. Therefore, we generalize these results to mean that small body sizes in spiders (not only in males) associate positively with higher species richness. This generalization allows for more explanatory power.

Even though the RF results unequivocally point towards minimum male body size to best predict species richness (Fig. 1), MCA reveals additional details regarding other predictor variables. Namely, a broad geographic distribution (range 5) shows a similar profile to high species richness, as well as small body size, in the two dimensions of the MCA (see their clustering in the upper left quarter of Fig. 4; also Fig. 5a–c and Fig. 6). On the other hand, smaller geographic ranges (2, 3, and 4) are associated with lower species richness (Figs. 4, 5, and 6). Limited geographic ranges may facilitate extinction rates and consequently decrease diversity [70]. Extinction rates are even faster for organisms with a combination of restricted geographic range, lower fecundity, and bigger body size [71, 72]. This agrees with our pattern where spider genera with larger body size tend to be species poor, and have generally narrower geographic distributions. We interpret this recovered pattern to be consistent with the predictions of the hypothesis that extreme phenotypes might decelerate speciation and/or cause extinction [59].

The next pattern, recovered by the MCA, shows that phylogenetic rank could have some potential in predicting species richness, even if not detected by the RF. The group that we refer to as “Orthognatha” (this unites, in paraphyly, the clades Mesothelae, the most primitive branch of spiders, and Mygalomorphae) is phylogenetically older [73, 74], and species poorer compared with the “Haplogynae” and “Entelegyne” spider clades. The Entelegyne and Haplogynae categories, however, do not show significant differences in species richness, which likely reduces the RF predicting power. Combining those two categories into “Araneomorphae” (a true clade) would likely increase RF accuracy. On the other hand, combining these categories diminishes the total information within the data and might mask relationships of those variable categories with others, e.g., with max COI distances (Fig. 5).

The debate whether or not clade age has a direct effect on species richness is unresolved [3, 29, 31, 32, 75, 76]. The effect of the time-for-speciation might produce such conflicting results due to tendencies of research to focus too broadly on taxa of incomparable ranks [77]. Although older clade age alone should in theory increase species richness [29, 30], the relationship between speciation and extinction rates is much more delicate [3]. The combination of larger body size, longer generation time, and geographically restricted distribution of organisms all theoretically decrease species richness and counter the “time-for-speciation effect” [71, 72]. This latter combination of factors might be relevant for the observed pattern in our case. The genera within “Orthognatha” are geographically more restricted, generally bigger, have longer generation times, and are species poor compared to the Araneomorphae genera [73, 78–81]. However, the observed pattern does not imply causality but rather uncovers some predictive value for species richness in the phylogenetic ranks variable category.

Finally, genetic distances do not appear to be associated with either high or low species richness. While the RF does recover some predictive value in max COI distances, categorical COI distance data in MCA bear no correlation with species richness. Instead of the expected correlation of COI distances with species richness, we observe lower maximum COI interspecific distances in Entelegyne compared with the genera within Haplogyne and “Orthognatha” (Figs. 4 and 5d, e). It has been known that COI distances strongly depend on taxonomic groups and practices [82]. Therefore, COI distances might contain more information than we recover here.

Given our understanding of spider biology, we find it surprising that ballooning, a behavior associated with dispersal in spiders, cannot predict species richness. Many spider species with high dispersal abilities use ballooning to travel across large distances and to colonize remote islands [37, 63, 83, 84]. While prior works suggest that dispersal ability may shape biodiversity [8, 33, 35, 85–87], our results indicate that it cannot accurately predict species richness. However, the alternative is that the ballooning behavior per se is not a good proxy for organismal dispersal ability. It also seems surprising that sexual size dimorphism cannot predict species richness. A female-biased SSD is highly pronounced in certain groups of spiders, notably orbweavers [60]. Kuntner and Coddington [59] hypothesize that extreme phenotypes may represent evolutionary dead-ends. Although, as speculated above, size may fit this prediction, it seems that SSD as a derived ratio does not. Hence, the support for this hypothesis is equivocal.

## Conclusions

Our study pioneers machine learning analyses in deciphering the factors that associate with diversity patterns. Given the power of this methodology, it may be worthwhile to reassess the here detected patterns on larger datasets on organisms other than spiders. Caution aside, what emerges from our study on spiders is that small body size and wide geographic ranges both associate with high species diversity. Future studies ought to test if this can be considered a broader biological phenomenon.

## Methods

### Data acquisition

We assembled a dataset containing 45 spider genera and multiple attributes (predictor variables) that could potentially affect species richness (dependent variable). We categorized the predictor variables into four groups: morphological, genetic, geographic, and “other” (containing phylogenetic rank, presence of ballooning, foraging type, and sexual size dimorphism (SSD)). We targeted spider genera, those that had publicly available data from the above attributes, randomly. Moreover, we put an effort to select the genera that exhibited significant variation in predictor variables, as well as variation in species richness. Whenever possible, we ensured that variables of categorical data were approximately equally represented by the number of observations in each category (Additional file 2).

#### Morphological variables

We used body size information as a morphological predictor variable. We obtained the following data: (a) maximum female body size, represented by the largest species within a genus; (b) minimum female body size, represented by the smallest species within a genus; (c) maximum male body size, represented by the largest species within a genus; and (d) minimum male body size, represented by the smallest species within a genus. From those values, we calculated the average body sizes and variation in body sizes for males and females and for both sexes combined. This resulted in ten body size variable permutations for the analyses. We obtained body size information primarily from Araneae, Spiders of Europe database [88] and consulted the original literature for genera not represented in that database (see Additional file 2).

#### Genetic variables

We used genetic distances, calculated from COI data, as a genetic predictor variable. We data-mined BOLD systems or GenBank for all publicly available COI sequences per targeted genus. We then discarded those sequences that were shorter than 600 nucleotides and those without a species identification. We selected a single sequence per species to calculate pairwise distances in MEGA [89]. We used the K2P parameter and a pairwise deletion option to calculate the minimum, maximum, and mean interspecific (congeneric) genetic distances within each genus (Additional file 2).

#### Geographic variables

We formed four geographic predictor variables. First, we ranked the geographic range of each targeted spider genus. We used the information on species occurrences from the World Spider Catalogue (WSC) [56] and Global Biodiversity Information Facility (GBIF) [90] and classified genus geographic ranges with the following criteria: (rank 1) all species within the genus are distributed locally, e.g., within a single archipelago; (rank 2) all congeneric species are distributed within a single continent; (rank 3) all congeneric species are distributed between two continents; (rank 4) all congeneric species are distributed among three continents; and (rank 5) congeneric species occur on four or more continents, i.e., the genus is cosmopolitan. Second, we counted the single island endemic species within each genus [56] and calculated the percent congeneric single island endemics. Third, we counted the congeneric species whose occurrences are limited to a single country (excluding island countries from the previous step), and calculated the percent congeneric species with a limited distribution. Finally, we combined the percent of single island endemics and the percent of single country occurrences into the fourth geographic predictor, the percent of congeneric species with a “narrow range” (Additional file 2).

#### Other variables

We formed additional four predictor variables. We categorized genera into four phylogenetic ranks: (a) Mesothelae, (b) Mygalomorphae, (c) Haplogynae, and (d) Entelegynae. Those distinct spider clades of different evolutionary ages [73, 74] represent an approximation of a clade age predictor variable. However, after preliminary analysis, we combined the Mesothelae and Mygalomorphae clades into one group “Orthognatha” because separately, both classes were underrepresented by the number of data points. Although paraphyletic, the group “Orthognatha” is evolutionary the oldest, the Entelegynae is the youngest, and Haplogynae is intermediate. Entelegynae and Haplogynae clades together represent the Araneomorphae spiders (Additional file 2).

From the behavioral ecology field, we included the foraging type and the presence of ballooning dispersal as predictors. The type of foraging was classified as either a “trap” or a “cursorial.” The “trap” comprises prey capture by web or ambush, while a webless, active search for food determines the “cursorial” category. The presence of ballooning dispersal was classified as “yes” or “no” according to the review on spider ballooning [63] (Additional file 2).

The last predictor variable was the presence or absence of sexual size dimorphism (SSD). We calculated SSD from the average body size of a species within the genus. If the ratio between average female and male body sizes exceeded 1.5, we classified the genus as having species with SSD (“yes”); otherwise, we assumed such genus does not contain sexually size dimorphic species (“no”). As the literature takes a ratio of 2.0 already as extreme SSD [59], our arbitrarily chosen ratio of 1.5 already accounts of moderate (as well as extreme) SSD. We acknowledge that calculating SSD from a single species within a genus is likely to produce false negative results but we had to accept the restrictions that pertain to a large dataset (Additional file 2).

#### Species richness as the dependent variable

We obtained the total number of described species within each targeted genus from WSC [56]. We left species richness as a numerical dependent variable for the Random Forest (RF) regression models and categorized it for RF classification models as well as for multiple correspondence analyses (MCA). We used alternative definitions for species richness categories, ranging from two broad groups (“low” and “high”) to five narrower groups (“very high,” “high,” “medium,” “low,” “very low”), attempting to maintain all categories approximately equally represented by data points (Additional file 2).

Our methodology does not take into account taxonomic uncertainties, and thus, a potential caveat is that variation in taxonomic completeness among genera may bias results. To ameliorate this potential bias, our choice of analyzed genera was random. Furthermore, biases pertaining to unequally complete genus taxonomies are likely to be diminished by broad data categorization. The broader the species richness categories, the lower the impact of undescribed species.

### Analytical protocols

#### Random Forest

The power of Random Forest (RF) predictions is based on “mean decrease GINI,” an index that explains the predictive power of each variable in regression or classification [91]. The greater the Gini decrease, the greater role that predictor variable has [91, 92]. The importance of features under assessment can thus be ranked, providing an intuitive graphical interpretation (Fig. 1). RF’s performance when faced with multiple collinear variables in the dataset is usually superior to the more conventional regression models and other methods of multivariate statistics due to its non-parametric nature, random selection of features at each node creation, and recursive partitioning [93–95]. While RF should accurately identify the best predictor even among highly correlated variables, some variables that correlate with the best predictor might have artificially lowered importance index relative to the best predictor. Therefore, caution is advised if one is to interpret the relative importance among correlated variables [96, 97].

We used the randomForest package [98] in R [99] to construct ten RF models. The first six RF models classified species richness as two categories. We ran the first RF analysis using all 22 predictor variables. RF analyses 2–5 used a subset of variables, “morphological,” “geographic,” “genetic,” and other,” while the last RF analysis only contained a single best predictor for species richness from each of the previous categories (“important”). The RF model using the “important” predictor variables that are not collinear also minimizes any potential dilemma that might emerge from RF analyses of all predictor variables, of which some do exhibit a degree of collinearity. We performed RF classification with species richness variable split into three categories for the analyses 7 and 8, which used all predictor variables and “important” predictors, respectively. The two regression models of RF also used “all” and “important” predictor variables. The dataset for RF analyses contained a combination of binary, categorical, and numerical data. We transformed geographic ranges (1 to 5) from numerical into factor variable. The data was then randomly split into training (*n* = 32) and test datasets (*n* = 13) except for the regression models where a training dataset had to be larger (*n* = 40) to facilitate “learning.” We ran RF on the training dataset and optimized RF models by searching the optimal “mtry” and “ntree” values to reduce “out of the bag” error (OOB). Finally, each trained RF model’s accuracy was evaluated with the test dataset. See supporting materials (Additional file 3) for R script.

#### Managing randomness of RF analyses

Each analysis that employs machine learning algorithms such as RF inevitably leads to results of slightly different outcomes. The first and the most obvious reason is a random splitting of the data into the training and test datasets. Following this are the random feature selection at each node creation when searching for the best “mtry” and another random feature selection when running a RF analysis. To investigate our RF’s performance beyond a single random event that might, by chance, produce spurious results, we ran each of the ten RF analyses under ten different seed numbers in R (set.seed = 1 to 10), totaling 100 RF predictions. We then checked for the consistency of the predictions and selected the RF results with the lowest estimated OOB error from each analysis. For reproducibility of our RF analyses, we include the information on randomness as the seed numbers used in each analysis in the R script (Additional file 3).

#### Multiple correspondence analysis (MCA)

Following the RF analyses, we selected the best predictor from each group of variables. We further analyzed the relations between the selected predictors and species richness with multiple correspondence analysis (MCA). We used the FactoMineR package [100] in R to run and visualize MCA. All variables in MCA are required to be categorical; therefore, we assigned classes to minimum male body size and maximum COI genetic distance. Males smaller than 5 mm (*n* = 22) were labeled “small” while males larger than 5 mm (*n* = 22) were labeled “big” (Additional file 2). Similarly, we assigned the genera with maximum COI genetic distance 18% or higher (*n* = 24) into a “large” category and the genera with lower values (*n* = 20) into a “little” category (Additional file 2). With preliminary MCA analysis, we identified a single extreme outlier *Heptathela*, the only genus with a range 1. The presence of one or more outliers in MCA can dominate the interpretation of the axes [101]; therefore, we eliminated *Heptathela* and proceeded with the remaining 44 genera.

While our initial MCA used two categories for species richness, minimum male body size, and maximum COI distances, we performed additional five MCA analyses with alternative category definitions to serve as method sensitivity tests. Species richness and minimum male body size categories ranged from two to five and maximum COI distances ranged from two to three. As described above, we attempted to keep all categories approximately equally represented by data points (Additional file 2). Additional file 4 contains the R script that can be used to repeat, or alter our analyses with alternative categories.

#### Confidence ellipse overlaps in the MCA dimensions

To add to the visual interpretation of MCA, we plotted the most relevant confidence ellipses of variable categories on a single plot. Moreover, we calculated the proportions of overlaps among these confidence ellipses using spatstat:utils R package [102] (for details, see Additional file 4).

#### Spearman’s correlation analysis

Following numerous RF and MCA analyses, we identified minimum male body size as the one variable that is most associated with species richness. Therefore, we also performed a more established correlation analysis between minimum male body size and species richness in R. We first tested the data for normality using the Shapiro–Wilk test, then based on these results performed Spearman’s rank correlation (details in Additional file 5).

## Supplementary information

**Additional file 1.** combines ten supplementary figures that give further credibility to the result presented in the main text. Figs. S1 and S2 show the Random Forest classification results with three species richness categories. Figs. S3 and S4 show the Random Forest regression results. Figs. S5 to S9 show Multiple correspondence analyses using varying numbers of species richness, minimum male body size and maximum COI genetic distance categories. Fig. S10 shows the results of Spearman’s correlation between minimum male body size and species richness.

**Additional file 2.** contains all data, mined from public databases or extracted from primary literature, which was used in Random Forest and Multiple correspondence analyses. In this excel file all category definitions can be explored or even altered and reanalyzed. Sheet three of this excel file contains instructions and references.

**Additional file 3.** contains R script with all the information needed to recreate our Random Forest analyses. (R 14 kb)

**Additional file 4.** contains R script with all the information needed to recreate our Multiple correspondence analyses. (R 52 kb)

**Additional file 5.** contains R script with all the information needed to recreate our Spearman’s rank correlation. (R 1 kb)

## Data Availability

All data generated or analyzed during this study are included in this published article and its supplementary information files (Additional files 1, 2, 3, 4, and 5).

## Abbreviations

RF: Random Forest
MCA: Multiple correspondence analysis
OOB: Out of bag error
SSD: Sexual size dimorphism
WSC: World Spider Catalogue
