# Supplementary material for: Machine learning approaches identify male body size as the most accurate predictor of species richness
Source: BMC Biol. 2020 Aug 28;18:105. doi: 10.1186/s12915-020-00835-y (PMC7453550; doi:10.1186/s12915-020-00835-y)

Additional file for:

Machine learning approaches identify male body size as the most accurate predictor of species richness

*Klemen Čandek[1,2,3], Urška Pristovšek Čandek[2,3], Matjaž Kuntner[1,2,4,5]

Supplementary figures S1 – S4: Additional Random Forest analyses

Supplementary figures S5 – S9: Additional Multiple correspondence analyses

Supplementary figure S10: Spearman's rank correlation

Supplementary figure S1:

Random Forest classification. Dependent variable “species richness” with “high”, “medium” and “low” classes. All predictor variables included.

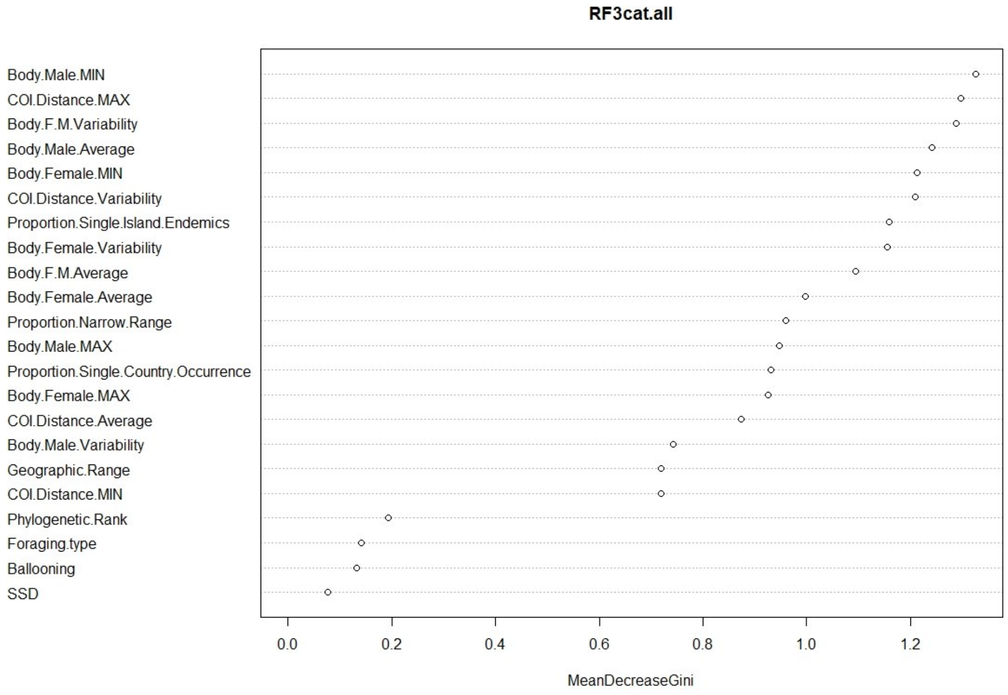

Supplementary figure S2:

Random Forest classification - dependent variable “species richness” with “high”, “medium” and “low” classes. Only the best predictor variable from each group of variables included.

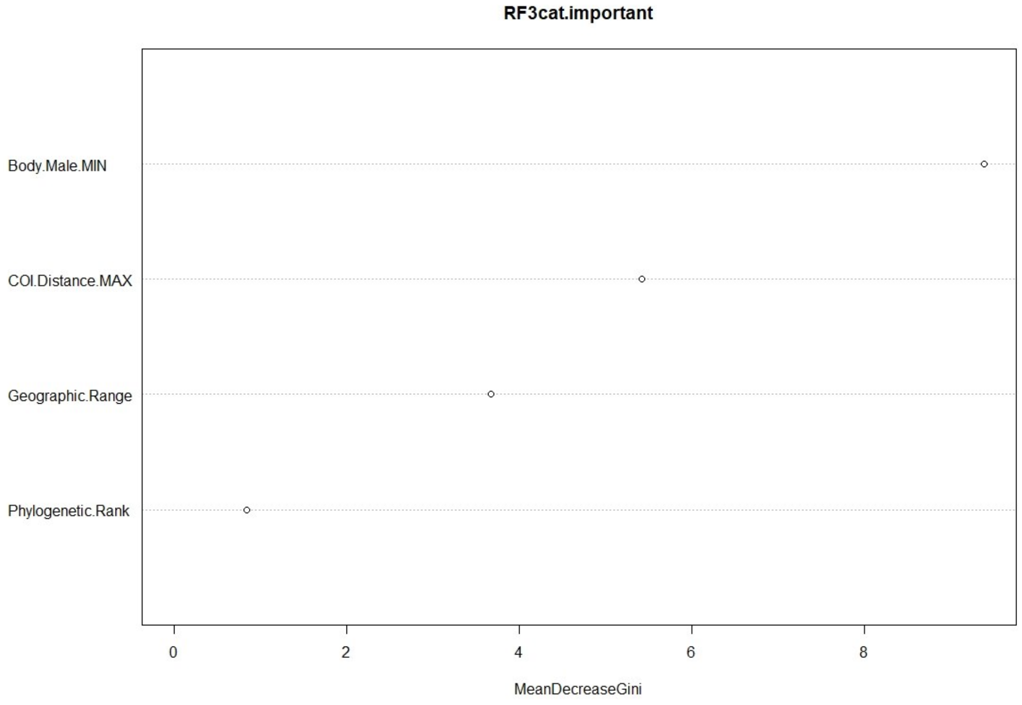

Supplementary figure S3:

Random Forest regression - dependent variable “species richness” as numeric variable. All predictor variables included.

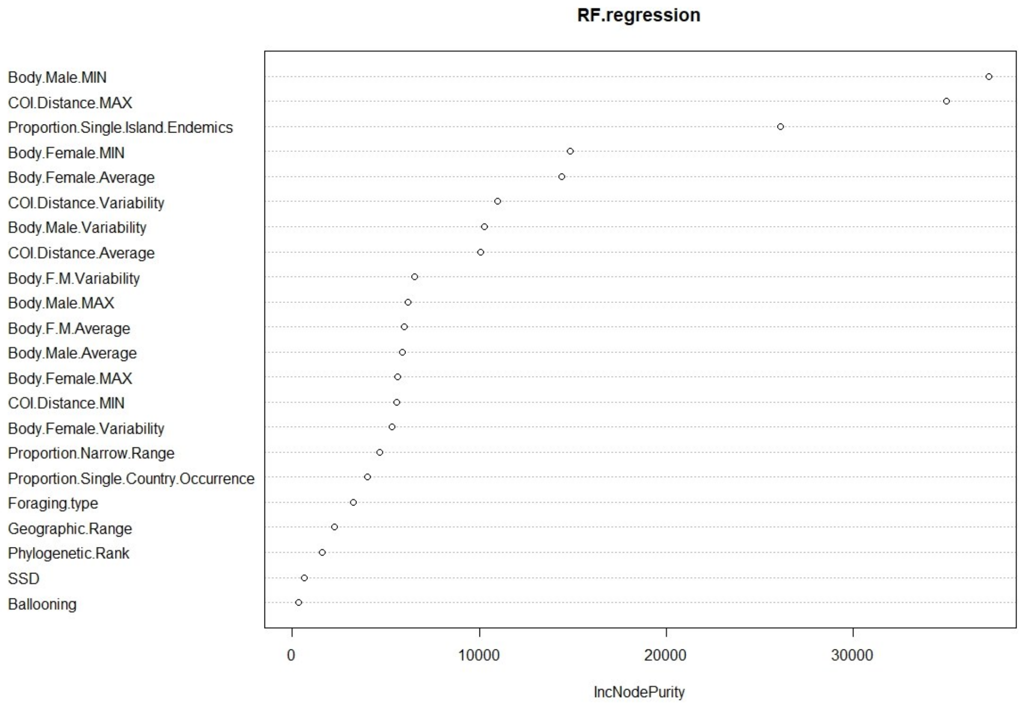

Supplementary figure S4:

Random Forest regression - dependent variable “species richness” as numeric variable. Only the best predictor variable from each group of variables included.

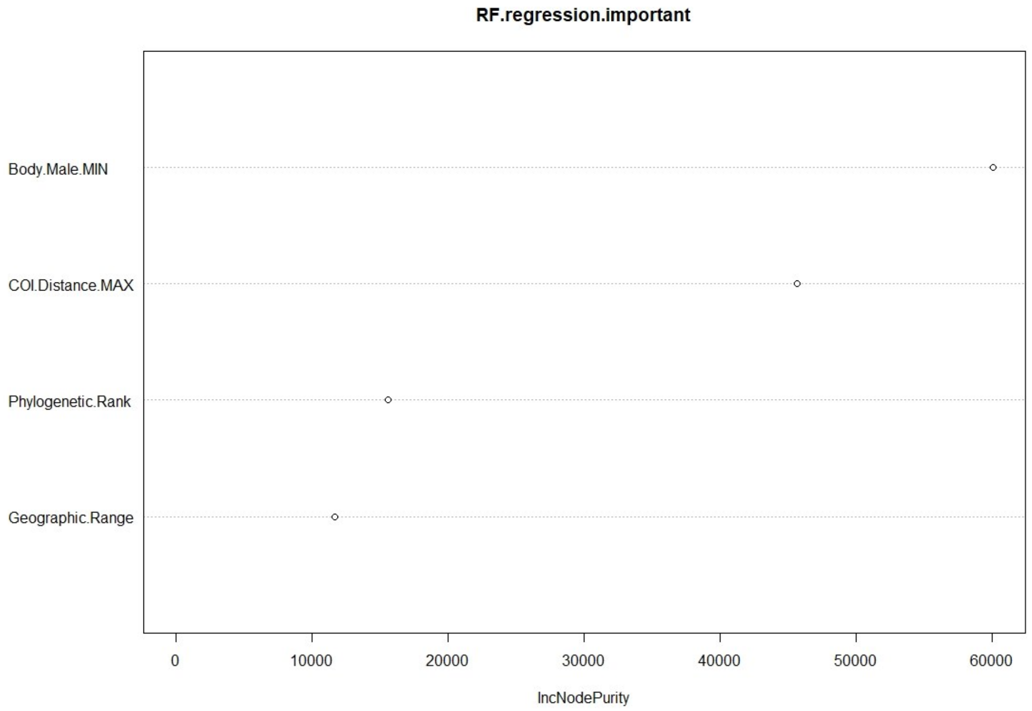

Supplementary figure S5:

MCA - Species richness as “high”, “medium” and “low”

A) Percentage of explained inertia in different dimensions

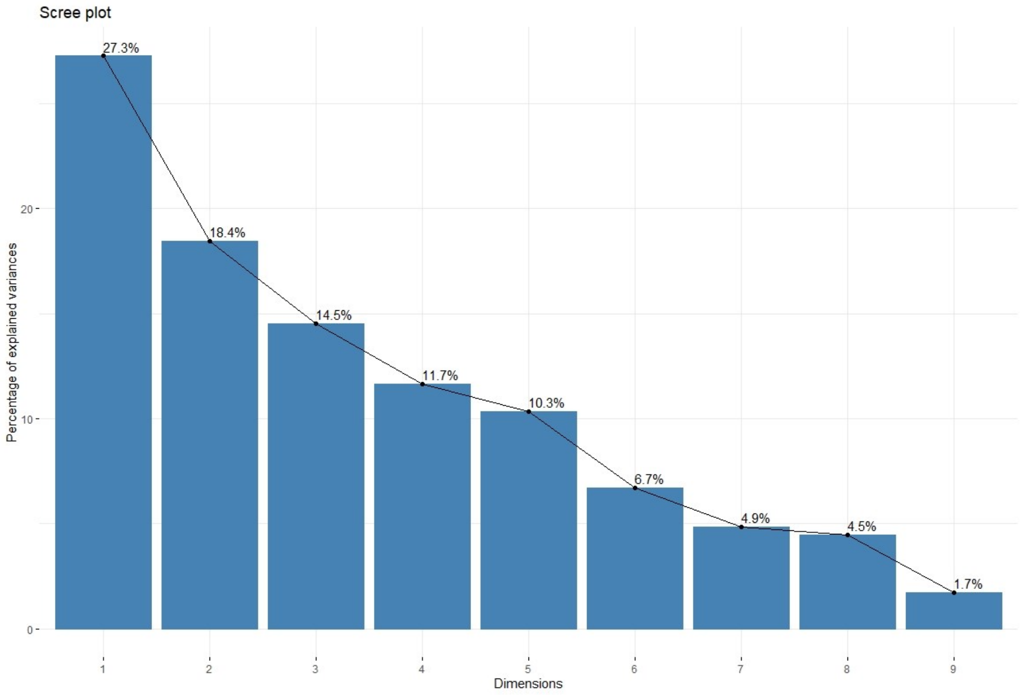

B) MCA factor map

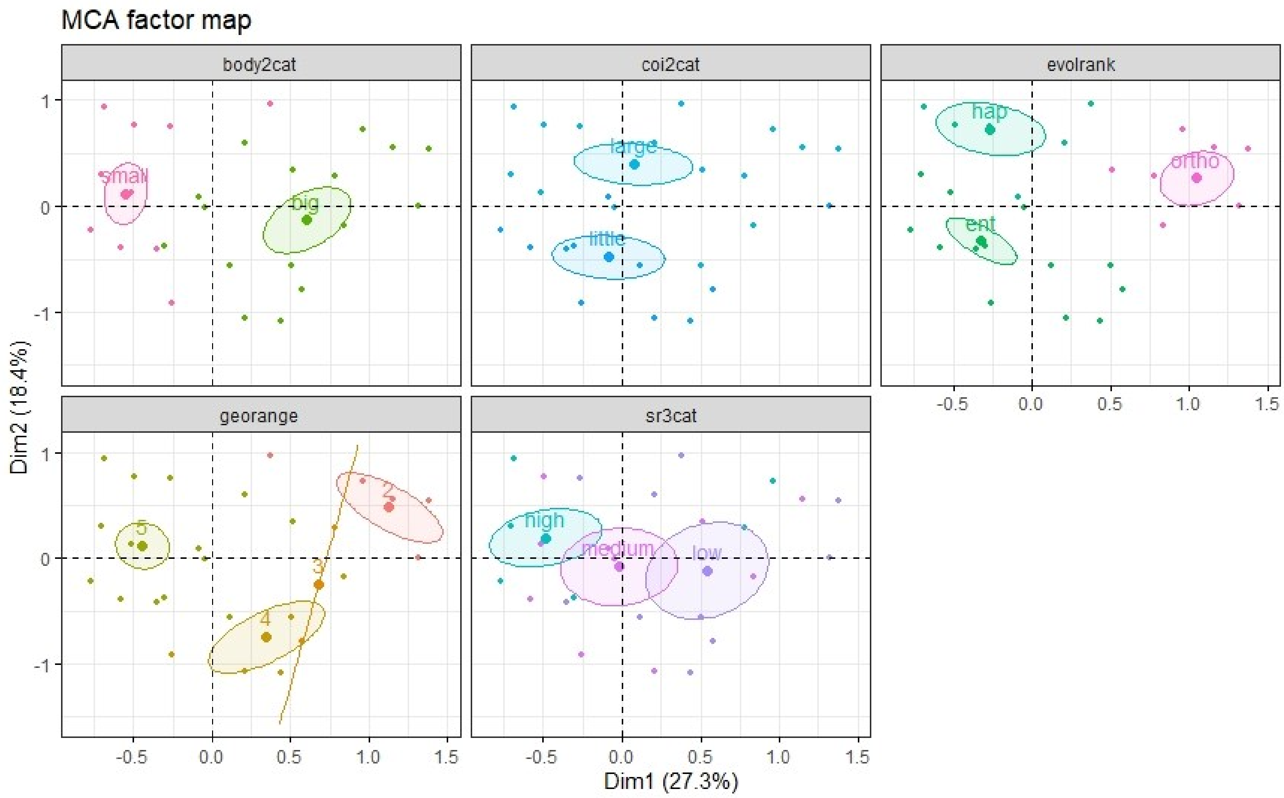

C) Confidence ellipse overlaps for male body sizes, geographic ranges and species richness

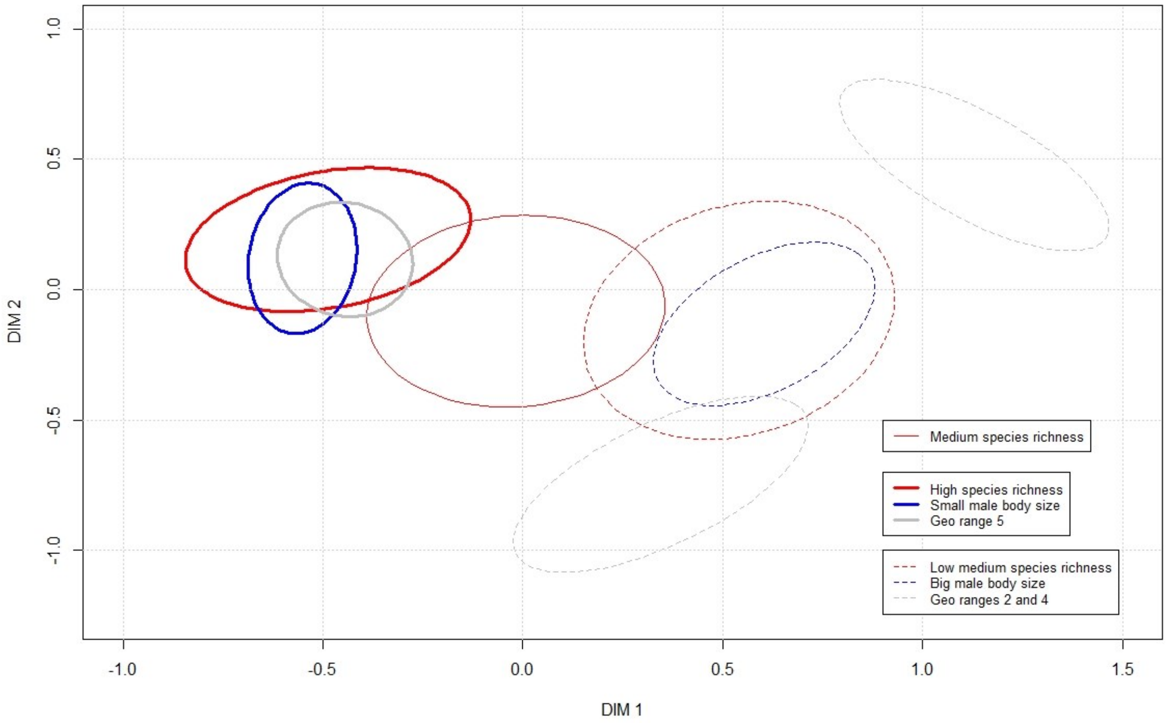

D) cos2 quality of representation for variable categories in different dimensions

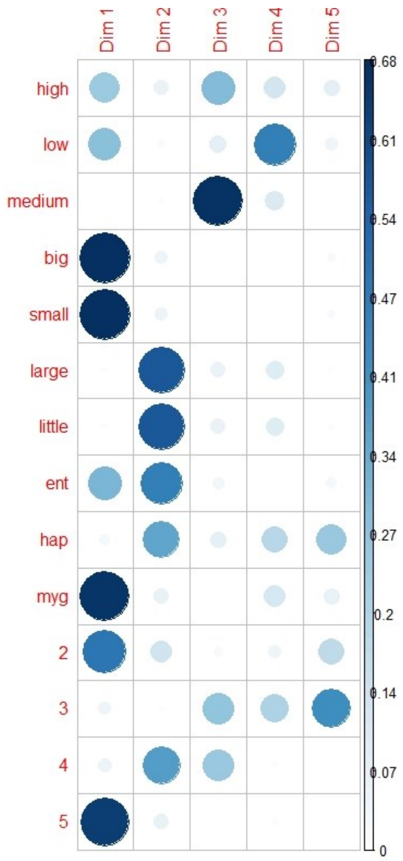

Important overlaps from panel "C":

37.16 % of the **high species richness** confidence ellipse area overlaps with the **small male body size** confidence ellipse

90.24 % of the **small male body size** confidence ellipse area overlaps with the **high species richness** confidence ellipse

34.28 % of the **high species richness** confidence ellipse area overlaps with the **geographic range 5** confidence ellipse

87.3 % of the **geographic range 5** confidence ellipse area overlaps with the **high species richness** confidence ellipse

59.44 % of the **small male body size** confidence ellipse area overlaps with the **geographic range 5** confidence ellipse

62.33 % of the **geographic range 5** confidence ellipse area overlaps with the **small male body size** confidence ellipse

44.11 % of the **low species richness** confidence ellipse area overlaps with the **big male body size** confidence ellipse

100 % of the **big male body size** confidence ellipse area overlaps with the **low species richness** confidence ellipse

Supplementary figure S6:

MCA - Species richness as “high”, “medium” and “low”, minimal male body size as “small”, “intermediate” and “big”

A) Percentage of explained inertia in different dimensions

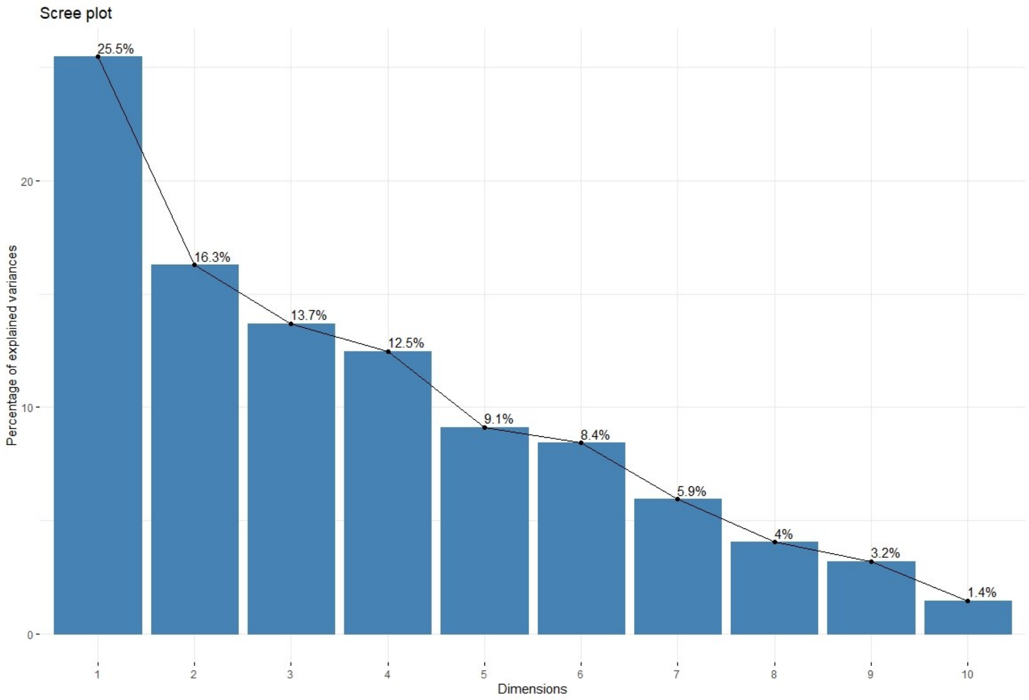

B) MCA factor map

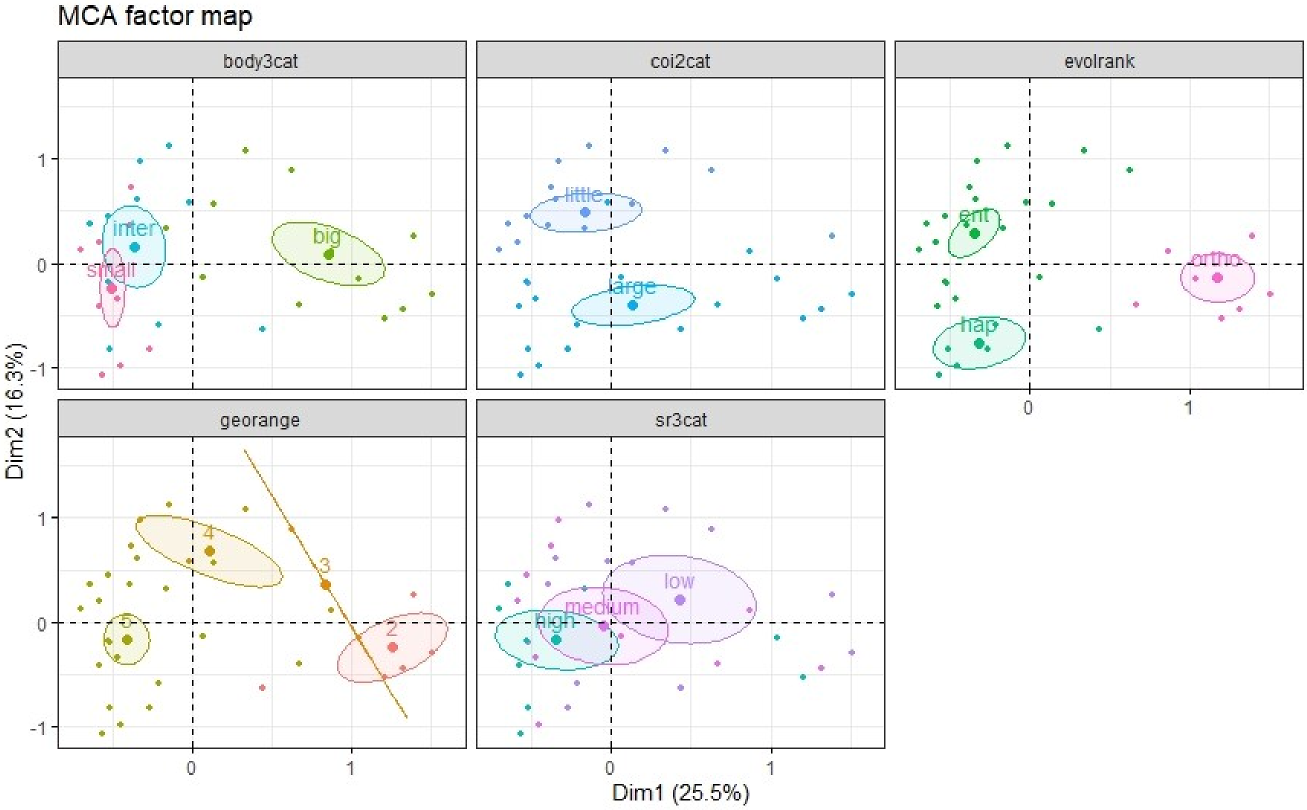

C) Confidence ellipse overlaps for male body sizes, geographic ranges and species richness

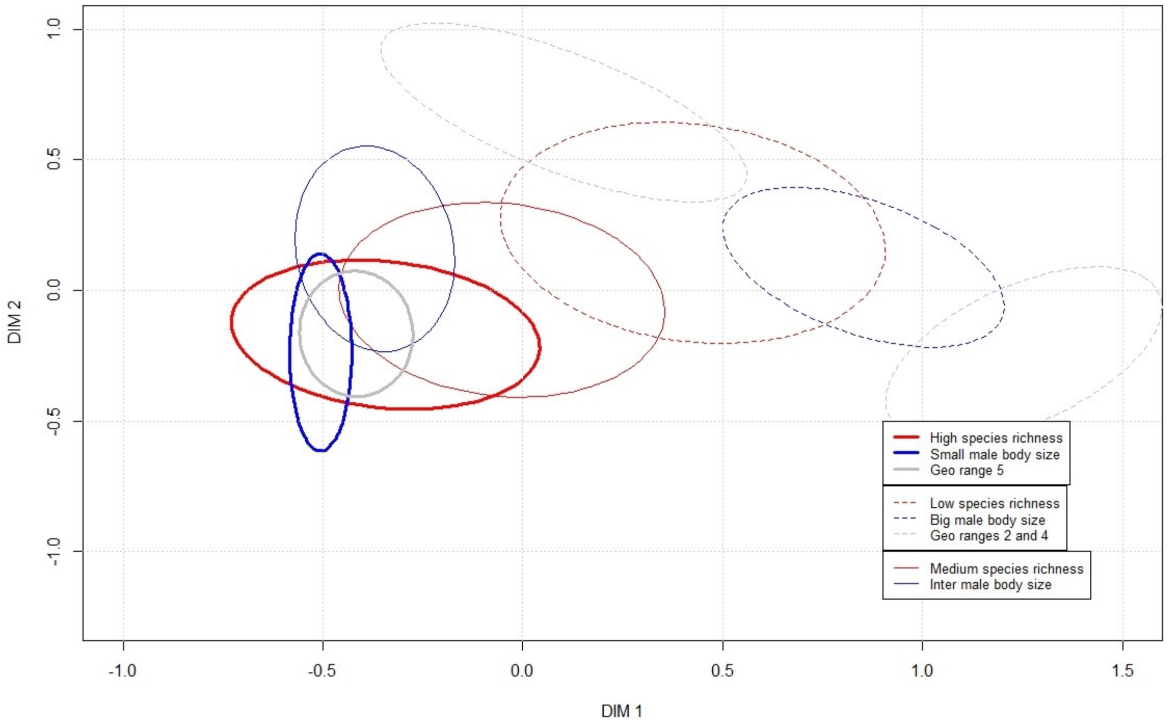

D) cos2 quality of representation for variable categories in different dimensions

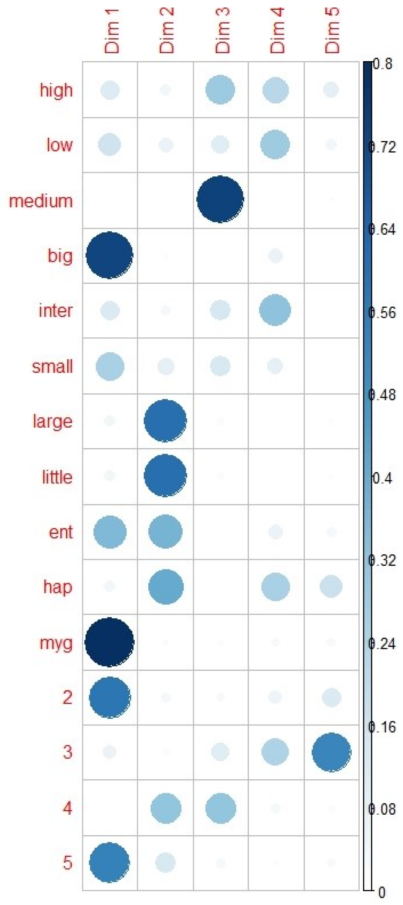

Important overlaps from panel "C":

20.26 % of the **high species richness** confidence ellipse area overlaps with the **small male body size** confidence ellipse

75.13 % of the **small male body** size confidence ellipse area overlaps with the **high species richness** confidence ellipse

31.41 % of the **high species richness** confidence ellipse area overlaps with the **geographic range 5** confidence ellipse

100 % of the **geographic range 5** confidence ellipse area overlaps with the **high species richness** confidence ellipse

48.54 % of the **small male body size** confidence ellipse area overlaps with the **geographic range 5** confidence ellipse

41.68 % of the **geographic range 5** confidence ellipse area overlaps with the **small male body size** confidence ellipse

24.61 % of the **low species richness** confidence ellipse area overlaps with the **big male body size** confidence ellipse

52.6 % of the **big male body size** confidence ellipse area overlaps with the **low species richness** confidence ellipse

25.04 % of the **medium species richness** confidence ellipse area overlaps with the **intermediate male body size** confidence ellipse

48.37 % of the **intermediate male body size** confidence ellipse area overlaps with the **medium species richness** confidence ellipse

Supplementary figure S7:

MCA - Species richness as “high”, “medium” and “low”; minimal male body size as “small”, “intermediate” and “big”; maximal COI genetic distances as “little”, “medium” and “large”

A) Percentage of explained inertia in different dimensions

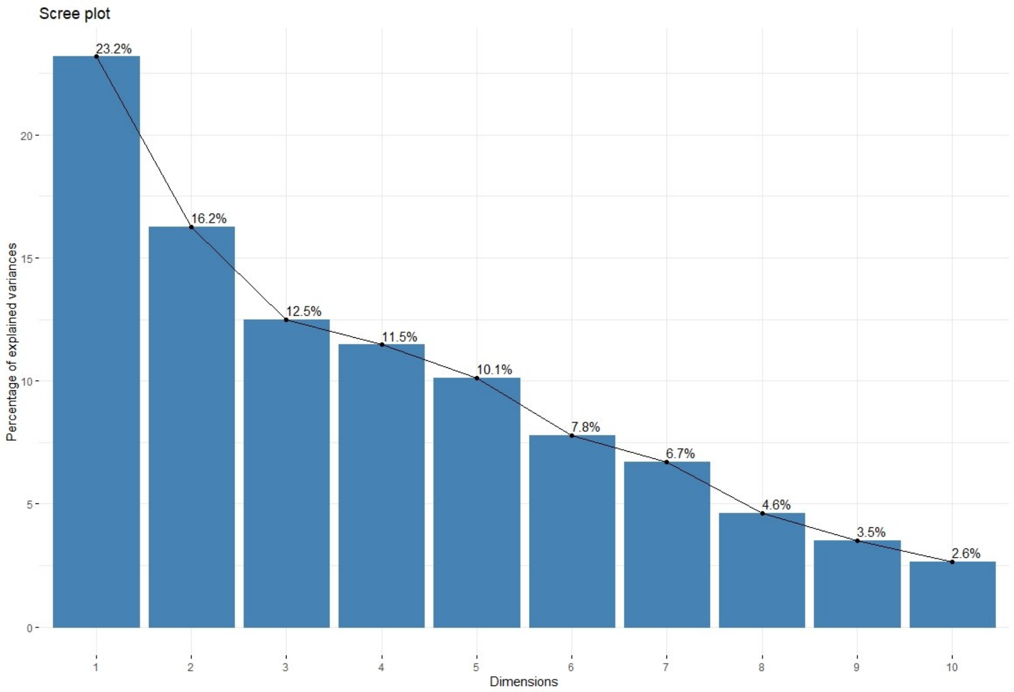

B) MCA factor map

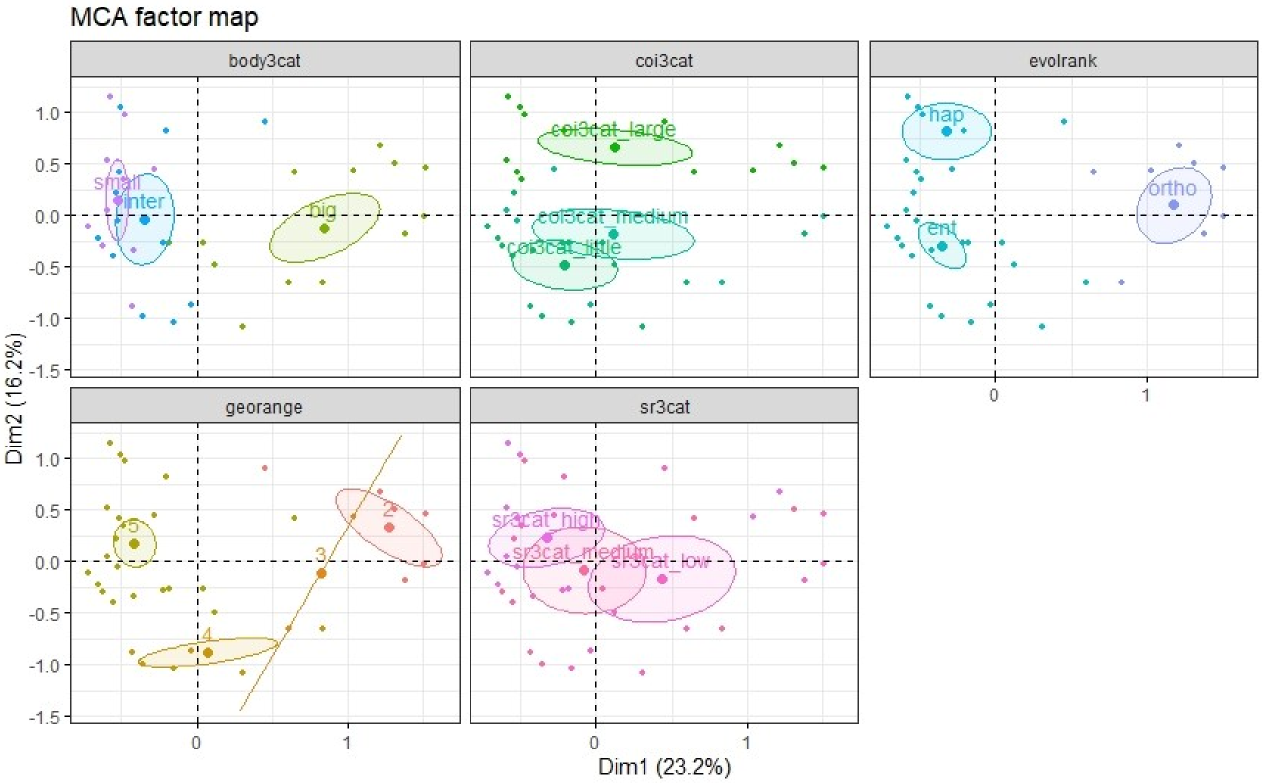

C) Confidence ellipse overlaps for male body sizes, geographic ranges and species richness

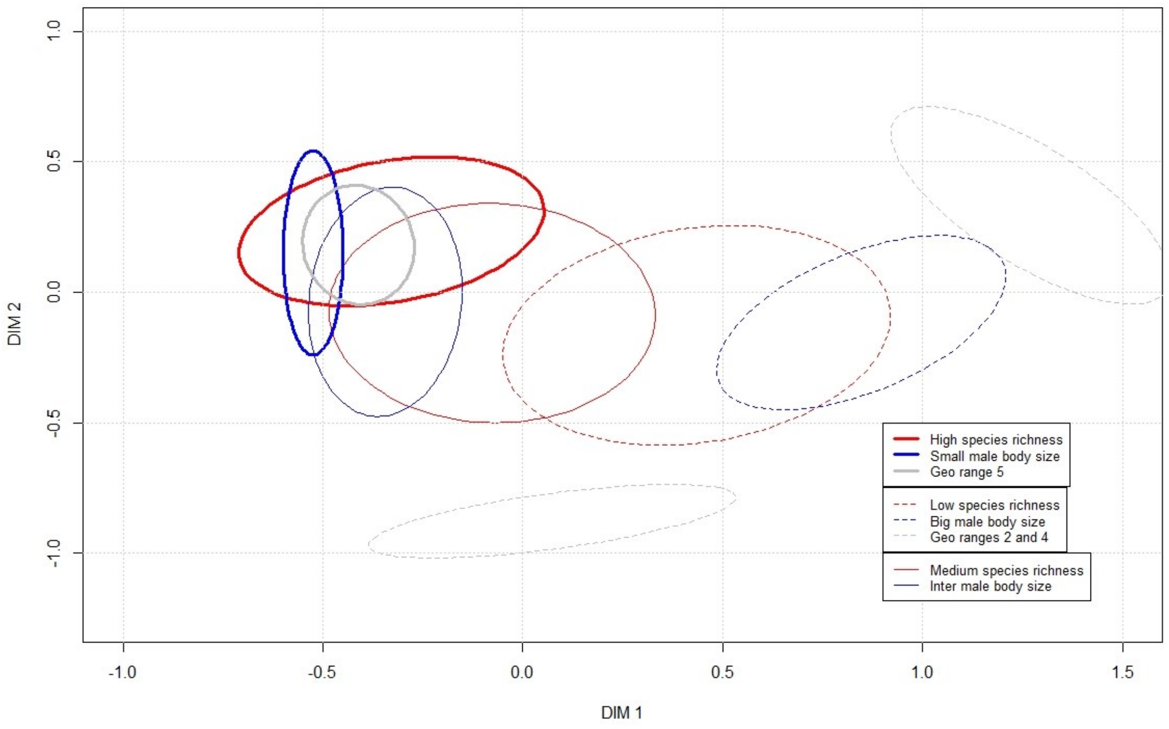

D) cos2 quality of representation for variable categories in different dimensions

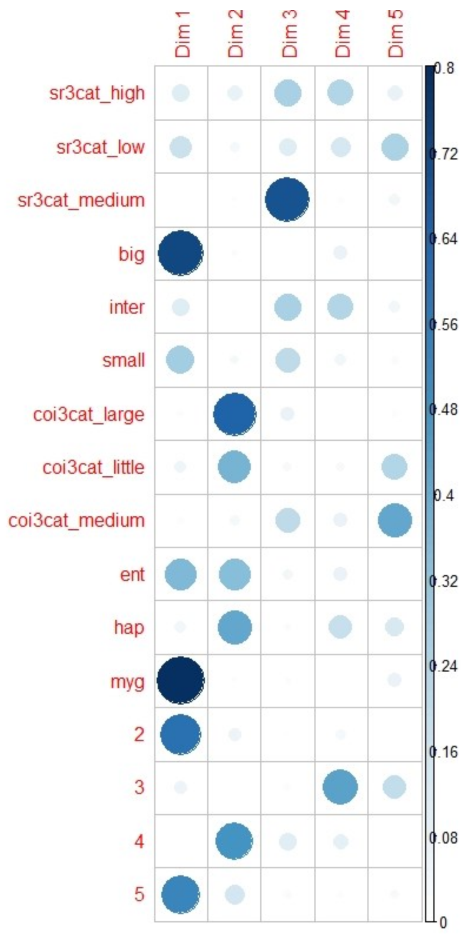

Important overlaps from panel "C":

19.65 % of the **high species richness** confidence ellipse area overlaps with **the small male body size** confidence ellipse

70.11 % of the **small male body size** confidence ellipse area overlaps with the **high species richness** confidence ellipse

30.51 % of the **high species richness** confidence ellipse area overlaps with the **geographic range 5** confidence ellipse

100 % of the **geographic range 5** confidence ellipse area overlaps with the **high species richness** confidence ellipse

33.44 % of the **small male body size** confidence ellipse area overlaps with the **geographic range 5** confidence ellipse

30.72 % of the **geographic range 5** confidence ellipse area overlaps with the **small male body size** confidence ellipse

28.23 % of the **low species richness** confidence ellipse area overlaps with the **big male body size** confidence ellipse

55.31 % of the **big male body size** confidence ellipse area overlaps with the **low species richness** confidence ellipse

33.34 % of the **medium species richness** confidence ellipse area overlaps with the **intermediate male body size** confidence ellipse

68.04 % of the **intermediate male body** size confidence ellipse area overlaps with the **medium species richness** confidence ellipse

Supplementary figure S8:

MCA - Species richness as “very high”, “high”, “low” and “very low”; minimal male body size as “very small”, “small”, “big” and “very big”; maximal COI genetic distances as “little” and “large”

A) Percentage of explained inertia in different dimensions

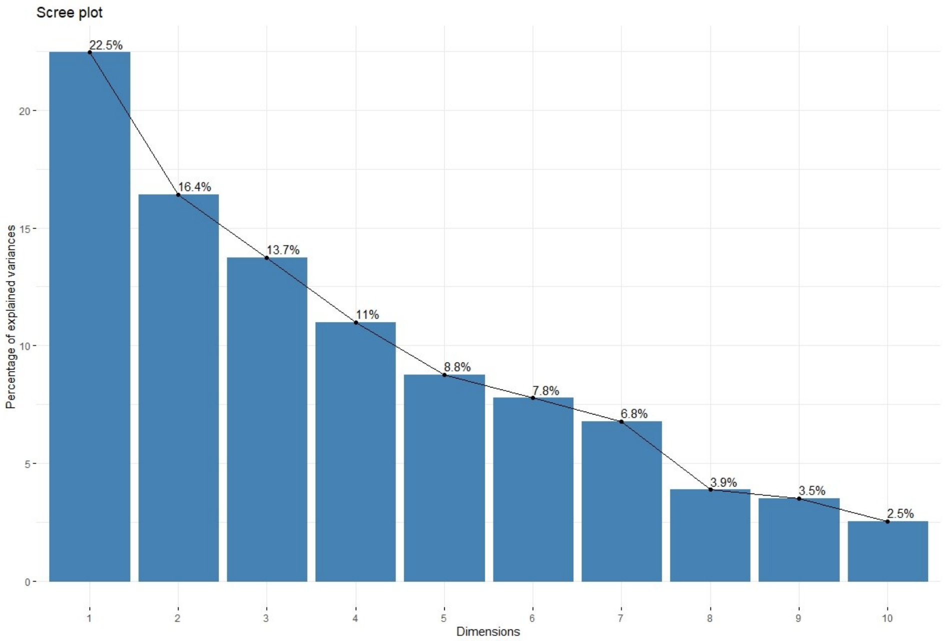

B) MCA factor map

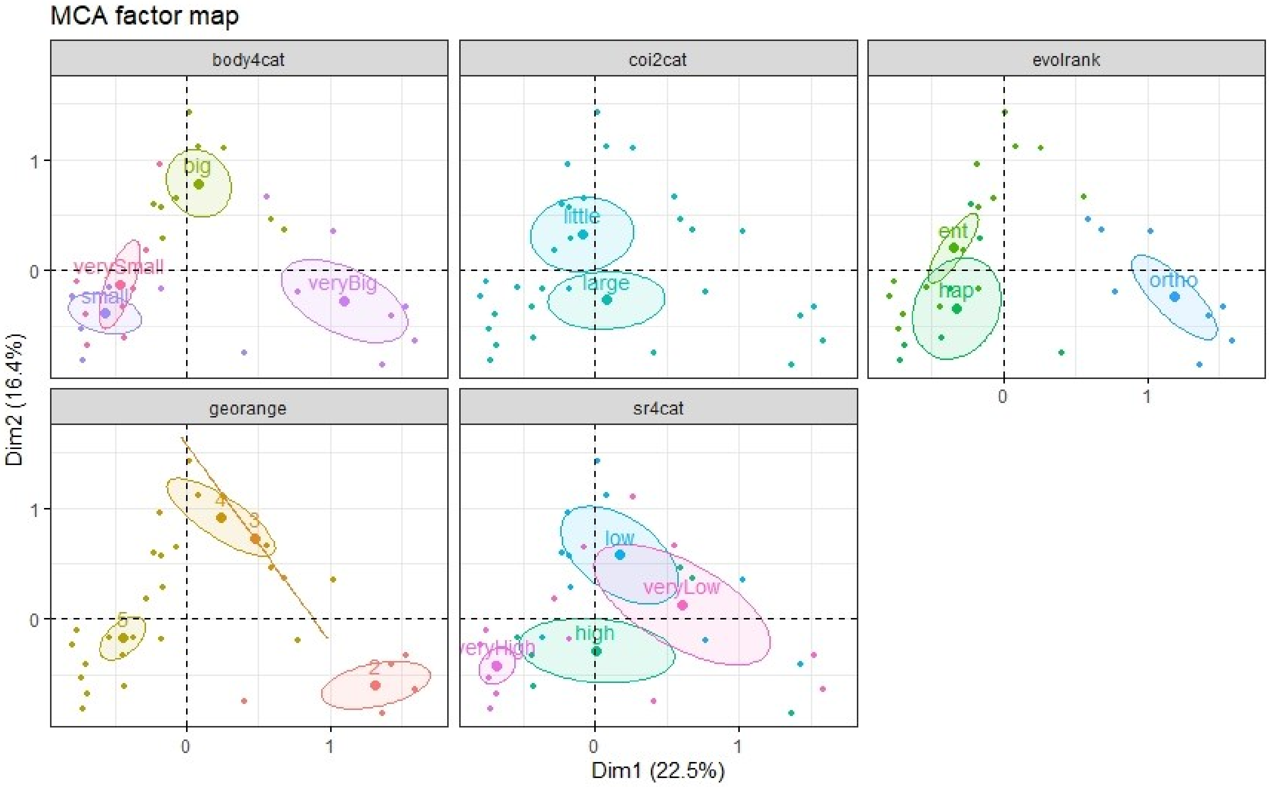

C) Confidence ellipse overlaps for male body sizes, geographic ranges and species richness

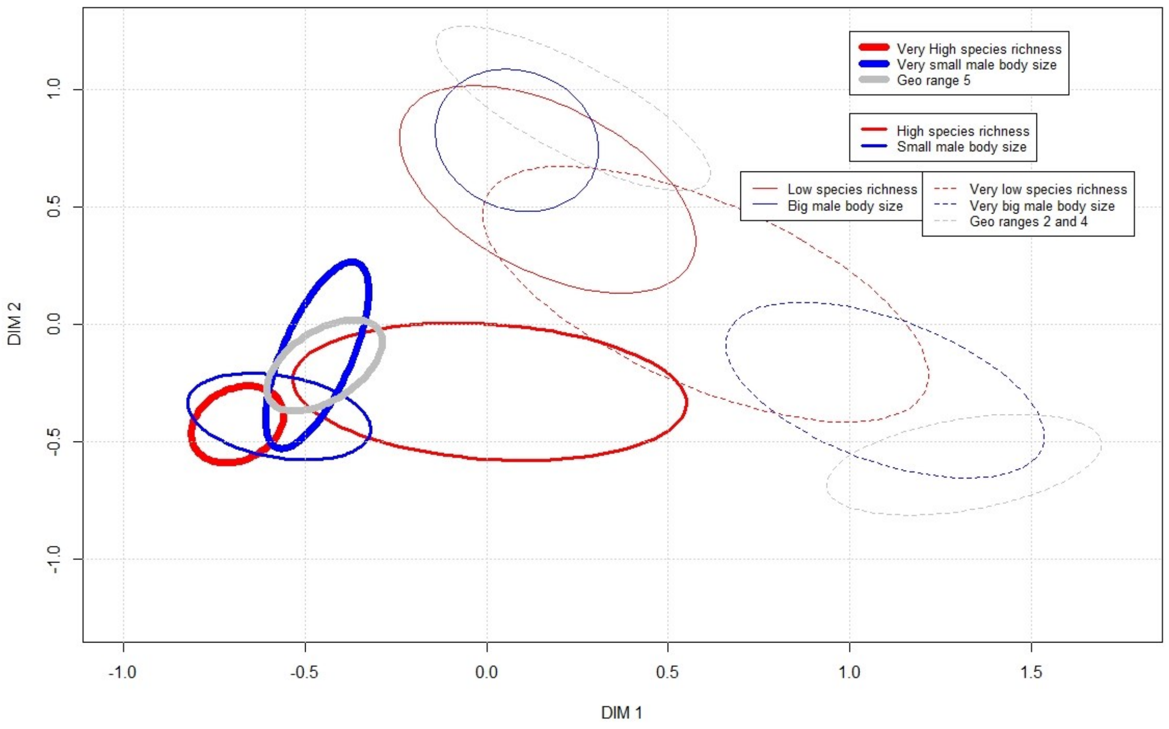

D) cos2 quality of representation for variable categories in different dimensions

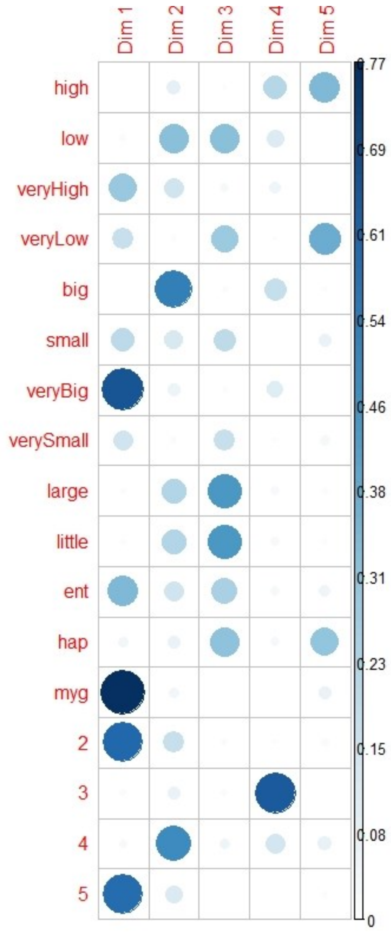

Important overlaps from panel "C":

77.76 % of the **very high species richness** confidence ellipse area overlaps with the **small male body size** confidence ellipse

35.44 % of the **small male body** size confidence ellipse area overlaps with the **very high species richness** confidence ellipse

12.14 % of the **very high species richness** confidence ellipse area overlaps with the **very small male body** size confidence ellipse

6 % of the **very small male body** size confidence ellipse area overlaps with the **very high species richness** confidence ellipse

50.88 % of the **very small male body** size confidence ellipse area overlaps with the **geographic range 5 confidence** ellipse

78.65 % of the **geographic range 5** confidence ellipse area overlaps with the **very small male body size** confidence ellipse

38.29 % of the **low species richness** confidence ellipse area overlaps with the **big male body size** confidence ellipse

88.45 % of the **big male body size** confidence ellipse area overlaps with the **low species richness** confidence ellipse

28.24 % of the **very low species richness** confidence ellipse area overlaps with the **very big male body size** confidence ellipse

52 % of the **very big male body size** confidence ellipse area overlaps with the **very low species richness** confidence ellipse

Supplementary figure S9:

MCA - Species richness as “very high”, “high”, “medium”, “low” and “very low”; minimal male body size as “very small”, “small”, “intermediate”, “big” and “very big”; maximal COI genetic distances as “little” and “large”

A) Percentage of explained inertia in different dimensions

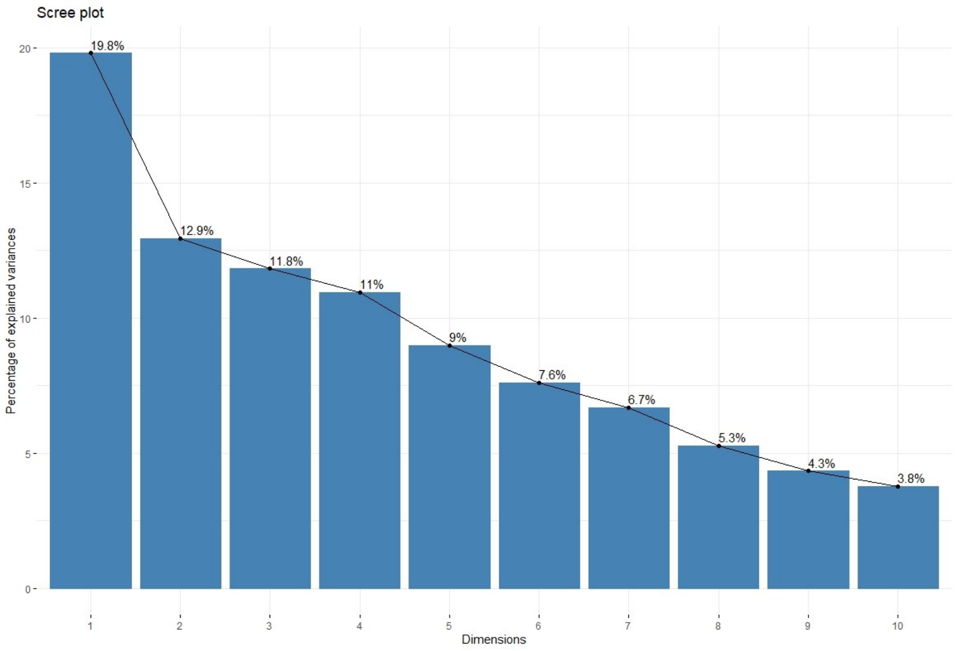

B) MCA factor map

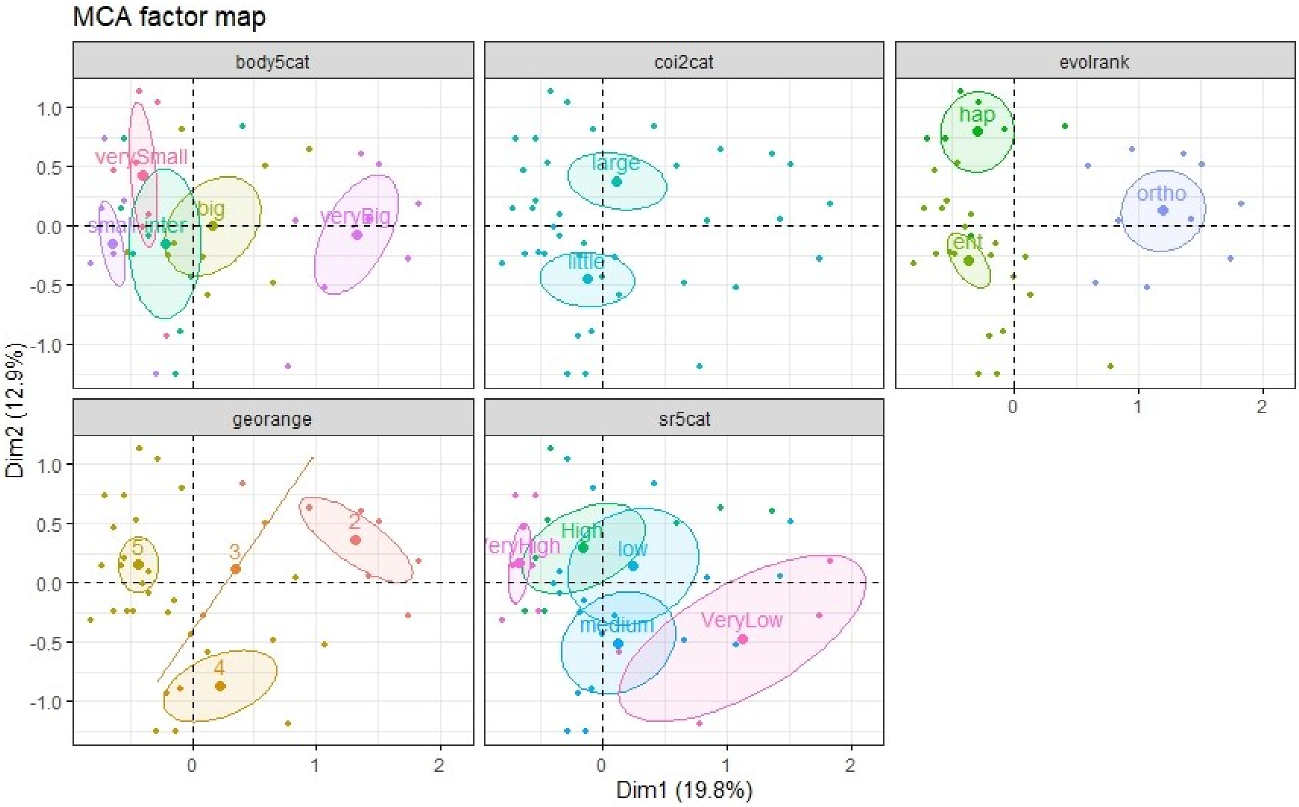

C*) Confidence ellipse overlaps for male body sizes, geographic ranges and species richness

*confidence ellipse overlaps not shown - too many object on a single plot obscure clarity. Overlaps can be calculated (and drawn) using our R script in Additional file 4.

D) cos2 quality of representation for variable categories in different dimensions

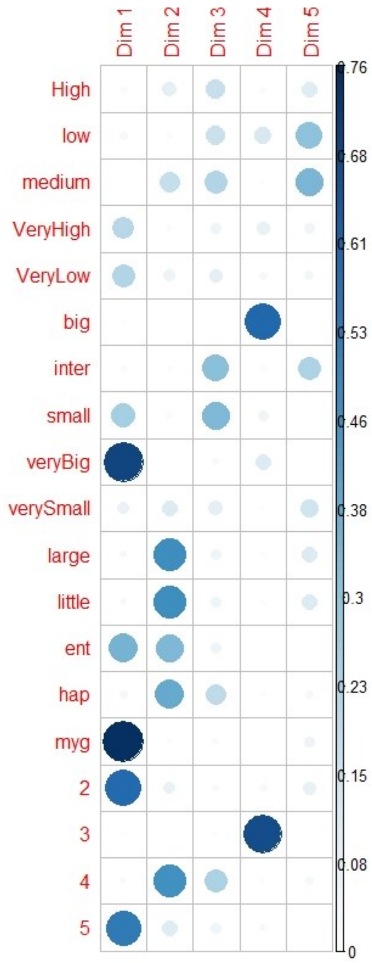

Supplementary figure S10:

Spearman's correlation of species richness and small male body size with normality tests

A) qqplot and Shapiro-Wilk normality test for species richness

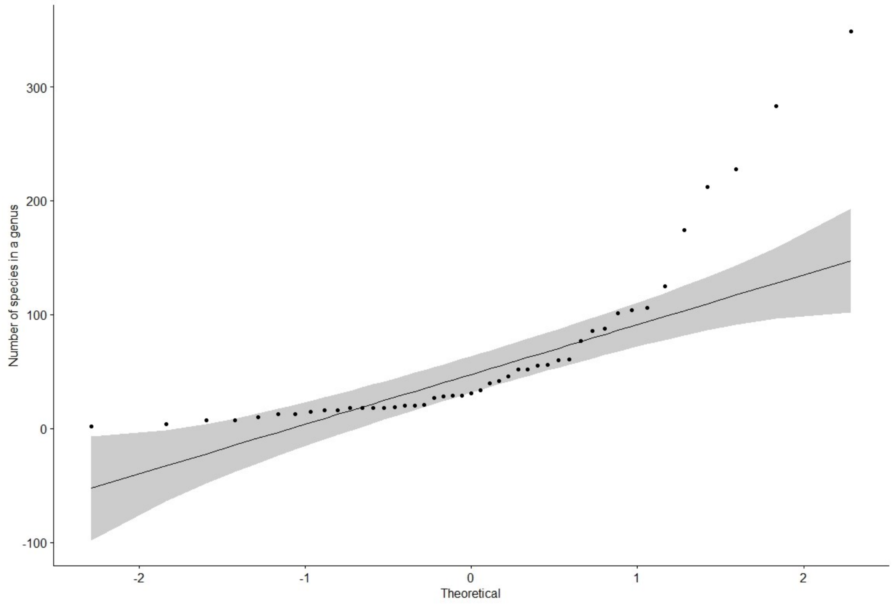

W = 0.7141;
p-value = 6.17e-08

B) qqplot and Shapiro-Wilk normality test for minimum male body size

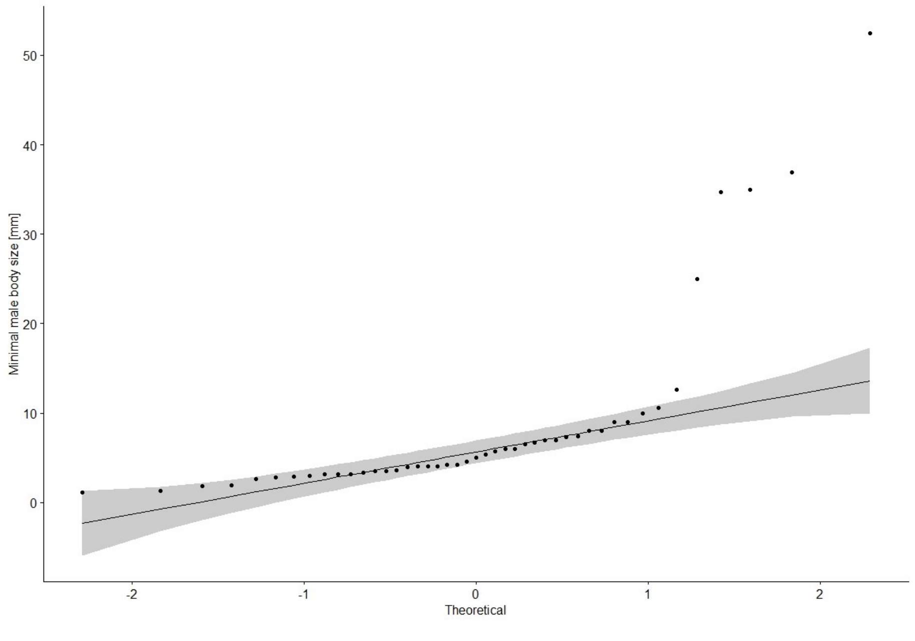

W = 0.59706;
p-value = 9.015e-10

C) Spearman correlation between minimal male body size and species richness

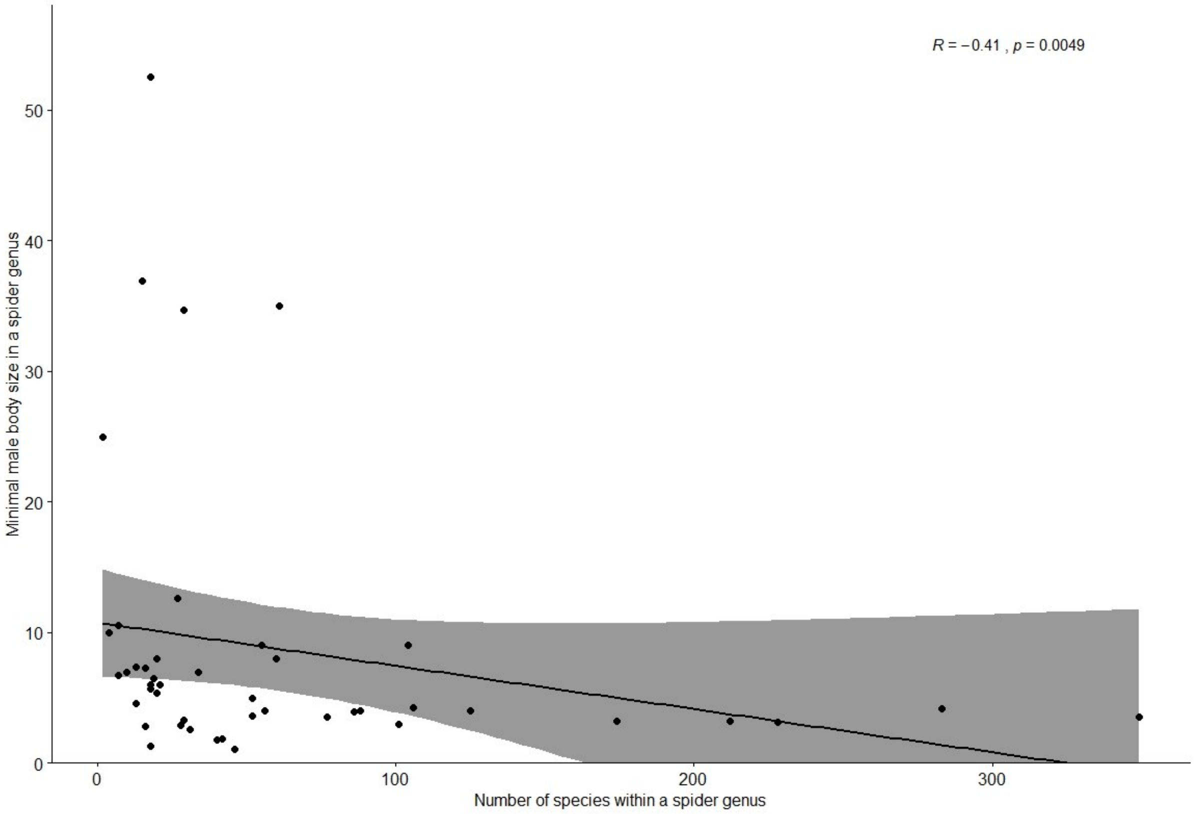

Supplement: Supplementary file 1 — Additional file 1. combines ten supplementary figures that give further credibility to the result presented in the main text. Figs. S1 and S2 show the Random Forest classification results with three species richness categories. Figs. S3 and S4 show the Random Forest regression results. Figs. S5 to S9 show Multiple correspondence analyses using varying numbers of species richness, minimum male body size and maximum COI genetic distance categories. Fig. S10 shows the results of Spearman’s correlation between minimum male body size and species richness. [file 12915_2020_835_MOESM1_ESM.pdf]
